# Integrated Chemical Profiling and Biological Assessment of *Melittis melissophyllum* L. (Lamiaceae): Antioxidant, Antimicrobial and Cytotoxic Activities

**DOI:** 10.3390/antibiotics15080742

**Published:** 2026-07-31

**Authors:** Daniela Benedec, Irina Ielciu, Emoke Pall, Adriana Dalila Criste, Adriana Cristina Urcan, Ilioara Oniga, Timea Henrietta Dezső (Bab), Ramona Flavia Burtescu, Daniela Hanganu

**Affiliations:** 1 Department of Pharmacognosy, Faculty of Pharmacy, “Iuliu Hațieganu” University of Medicine and Pharmacy, 400010 Cluj-Napoca, Romania; dbenedec@umfcluj.ro (D.B.); ioniga@umfcluj.ro (I.O.); bab.timea.henrietta@elearn.umfcluj.ro (T.H.D.); dhanganu@umfcluj.ro (D.H.); 2 Department of Pharmaceutical Botany, Faculty of Pharmacy, “Iuliu Hațieganu” University of Medicine and Pharmacy, 400337 Cluj-Napoca, Romania; 3 Department of Clinical Sciences, University of Agricultural Sciences and Veterinary Medicine Cluj-Napoca, 400001 Cluj-Napoca, Romania; emoke.pall@usamvcluj.ro; 4 Department of Microbiology and Immunology, Faculty of Animal Science and Biotechnologies, University of Agricultural Sciences and Veterinary Medicine Cluj-Napoca, 400001 Cluj-Napoca, Romania; adriana.criste@usamvcluj.ro (A.D.C.); adriana.urcan@usamvcluj.ro (A.C.U.); 5 PlantExtrakt Ltd., 407059 Cluj-Napoca, Romania; ramona.burtescu@plantextrakt.ro

**Keywords:** *Melittis melissophyllum*, polyphenols, antioxidant, antibacterial, antifungal, cytotoxic

## Abstract

**Background/Objectives: ***Melittis melissophyllum* L. (bastard balm) is a perennial herb belonging to the Lamiaceae family. This study investigated the phytochemical composition, antioxidant properties, antimicrobial activity, and cytotoxic effects of a 50% (*v*/*v*) ethanol–water extract of *M. melissophyllum*. **Methods:** The phytochemical profiles were characterized by spectrophotometric analysis, while individual polyphenolic compounds were identified and quantified using LC–MS. Antioxidant activity was assessed using the DPPH radical scavenging and FRAP assays. The cytotoxic effects were evaluated in normal human foreskin fibroblasts (BJ) and two human cancer cell lines, HCT-116 (colorectal carcinoma) and OVCAR-3 (ovarian adenocarcinoma). The antimicrobial efficacy of *M. melissophyllum* extracts was assessed against a panel of Gram-positive and Gram-negative bacterial strains, as well as a representative yeast species. **Results:** The results revealed a high content of polyphenolic compounds in the tested extract. Cytotoxicity assays demonstrated good tolerability toward normal cells while indicating inhibitory effects on tumor-derived cell lines. In addition, the extract exhibited limited but measurable strain-dependent antimicrobial activity. **Conclusions:** Collectively, these findings suggest that *M. melissophyllum* represents a promising source of bioactive compounds with potential medicinal applications.

## 1. Introduction

*Melittis melissophyllum* L. (bastard balm) belongs to the Lamiaceae family and is the only member in its genus. This herbaceous perennial commonly grows along woodland margins, in shaded environments, at forest borders, and on calcium-rich soils at low to moderate altitudes throughout central and southern Europe, including Romania’s Carpathian region [1,2,3,4]. The species is recognizable by leaves that are petiolate to subsessile, arranged in a decussate pattern, ovate to heart-shaped, and toothed along the edges; rubbing the leaves releases a coumarin-like fragrance. Flowers appear in white, pink, or purple shades and are generally clustered at the tips of the flowering stems. Its fruit takes the form of a four-part schizocarp [4,5,6]. It blooms from late spring into early summer, during which it supports biodiversity in the forest understory and provides nectar for pollinating insects [4,7]. Although *M. melissophyllum* does not currently hold status as an official botanical drug, it appeared in past editions of the French Pharmacopoeia and has historically been used in traditional medicines, particularly within Italy and Serbia [8].

Within the wider Lamiaceae family, *M. melissophyllum* stands out for its distinctive volatile emissions; notably, it produces unusual non-terpenoid compounds, including 1-octen-3-ol, which plays an ecological role in drawing specific insects and shaping overall scent characteristics [5,9,10,11]. Recent reviews on Lamiaceae aerobiology address this compound while highlighting the glandular trichomes and essential oil chemistry characteristic of the family [7]. It is well documented that Lamiaceae members serve as abundant sources of phenolic acids, flavonoids, and iridoid glycosides—compounds that frequently contribute to antimicrobial and antiproliferative properties [7,12,13]. Defining the chemical composition of *M. melissophyllum* may aid in creating standardized plant-based preparations or identifying candidates for pharmaceutical development. Taxonomically and phytochemically, two subspecies are recognized—*M. melissophyllum* subsp. *melissophyllum* and *M. melissophyllum* subsp. *albida*—with research from Italy revealing chemical distinctions (in both essential oil composition and iridoid content) between them, pointing to intraspecific chemical variation that may parallel morphological differences [5,14,15,16].

Traditionally, the species found a place in European herbal medicine for its soothing, wound-healing, antiseptic, antispasmodic, and digestive-supporting properties [4,8,10,17]. Despite this traditional use, comprehensive phytochemical and bioactivity research on *M. melissophyllum* remains limited compared to better-studied Lamiaceae genera like *Salvia*, *Thymus*, and *Melissa* [17]. Ethnobotanical documentation from several areas, including Romania and Italy, describes the use of both fresh and dried leaves in calming teas and regional treatments for nervous system and digestive complaints [5,16]. At present, the plant finds application in flavoring alcoholic beverages and tobacco products [4].

Extensive phytochemical investigations of *M. melissophyllum* have revealed numerous bioactive constituents, including essential oils, flavonoids, phenolic acids, and iridoid glycosides [3,4,5,6,8,9,18,19]. The species’ leaves harbor a broad range of phenolic compounds, among which flavonoids and coumarins represent major components. According to Szymborska-Sandhu and colleagues, the aerial portions of bastard balm accumulate substantial phenolic content—particularly flavonoids like luteolin, apigenin, quercetin, myricetin, and kaempferol, alongside phenolic acids such as chlorogenic, caffeic, and ferulic acids—as well as coumarins, which serve as an important quality indicator for the species. The phytochemical makeup of this species is notably shaped by environmental and developmental conditions, including shade exposure, light levels, seasonal timing, and plant maturity; full sun exposure enhances phenolic and flavonoid accumulation, whereas shaded conditions favor coumarin production—findings that have practical implications for cultivation and harvesting practices aimed at optimizing specific phytochemicals [4,6]. By contrast, essential oil yield tends to be minimal and its composition inconsistent, with characteristic input from non-terpenoid volatiles seen across Lamiaceae species. Key volatile components include citronellal, β-caryophyllene, and germacrene-D [5,18] along with 1-octen-3-ol, which imparts the plant’s characteristic mushroom-like scent [10,11,16]. Regarding iridoids, compounds like harpagide and related derivatives can be produced in greater quantities through in vitro shoot culture systems when subjected to elicitation treatments (such as methyl jasmonate, ethephon, or L-phenylalanine) combined with optimized growth media. Work by Skrzypczak-Pietraszek and colleagues showed that agitated shoot cultures, when elicited and supplied with precursors, yielded elevated levels of harpagide and 8-*O*-acetyl-harpagide, demonstrating a biotechnological strategy for boosting particular iridoids beyond what is achievable with field-cultivated plants [19]. Although much existing research emphasizes antioxidant capacity as the main pharmacological focus, the plant’s traditional applications—encompassing antispasmodic, digestive, sedative, and anxiolytic effects—have been partly attributed to its coumarin and flavonoid content, given these compounds’ established antioxidant, anti-inflammatory, and neuromodulatory activities [17,18].

There is robust evidence for antioxidant and related pharmacological properties, but translational and clinical confirmation remains limited; attention to standardization, pharmacokinetics, and safety is essential for translating these findings into approved therapies or validated nutraceuticals. The in vitro and in vivo data, together with traditional medical uses, reinforce the plant as a promising candidate for further pharmacological development. The present study aims to investigate the phytochemical composition and the in vitro antioxidant, antimicrobial and cytotoxic activities of *M. melissophyllum*, collected from the Romanian spontaneous flora.

## 2. Results

### 2.1. Spectrophotometric Analyses of Polyphenolic Metabolites and Antioxidant Activity

Table 1 presents the results obtained for the *M. melissophyllum* extract by spectrophotometric methods quantifying the total polyphenol content (TPC) expressed in mg gallic acid equivalents (GAE)/g dry weight (dw) vegetal product, the total flavonoid content (TFC) in mg rutin equivalents (RE)/g dw vegetal product and the concentration of caffeic acid derivatives (CADC) formulated in mg caffeic acid equivalents (CAE)/g dw vegetal product. Also, the same table (Table 1) presents the results obtained for the evaluation of the antioxidant potential by two classical methods: DPPH and FRAP.

Results show significant amounts of the three phenolic classes, which could be linked to the performed biological activity assays. Regarding the antioxidant activity, the IC_50_, calculated to determine the sample concentration required to inhibit 50% of DPPH radicals, was 0.18 ± 0.01 mg/mL, higher than the Trolox standard (0.012 ± 0.003 mg/mL). In this case, a lower IC_50_ value indicates a higher antioxidant activity. In the FRAP test, a higher value shows a higher antioxidant potential of the plant extract, the result being 1.060 ± 0.77 mg TE/mL. Statistical analysis of the DPPH assay results revealed a significant difference between the antioxidant activity of tested extract and the Trolox standard (*p* < 0.001), with the extract exhibiting lower radical scavenging activity than Trolox.

### 2.2. LC–MS Analysis of M. melissophyllum Extract

The results obtained by LC-MS analysis of the individual phenolic metabolites of the *M. melissophyllum* extract are shown in Table 2 and Figures S1–S17.

Obtained results show that chlorogenic acid was the main compound, accompanied by other phenolic acids important for biological effects, namely caffeic acid and salicylic acid. Flavonoids are represented by both glycosylated and free structures (aglycons), such as luteolin-7-*O*-glucoside, hyperoside, kaempferol, apigenin.

### 2.3. Antimicrobial Activity Assays

The *M. melissophyllum* extract was evaluated for antimicrobial activity against three Gram-positive pathogens, three Gram-negative pathogens, and one yeast strain. The highest antimicrobial activity was observed against *Bacillus cereus* ATCC 11778, with inhibition zones of 7.67 ± 0.11 mm. The lowest activity was recorded against *Staphylococcus aureus* MRSA ATCC 43300 (1.5 ± 0.15 mm) and *Pseudomonas aeruginosa* ATCC 27853 (2.50 ± 0.10 mm), both of which are known for their intrinsic resistance to antimicrobial agents. The solvent control, consisting of 50% ethanol in water (*v*/*v*; 50 µL), produced no detectable inhibition against any of the tested microorganisms (ZOI = 0 mm), confirming that the observed antimicrobial activity was attributable to the extract rather than to the extraction solvent (Table 3).

The MIC values of the *M. melissophyllum* extract ranged from 3.36 to 13.43 μg/μL. The highest MIC values were recorded for *Staphylococcus aureus* MRSA ATCC 43300 and *Pseudomonas aeruginosa* ATCC 27853 (13.43 μg/μL). Intermediate MIC values of 6.71 μg/μL were observed for *Escherichia coli* ATCC 25922 and *Salmonella enteritidis* ATCC 25928. *Bacillus cereus* ATCC 11778, *Staphylococcus aureus* ATCC 25923, and *Candida albicans* ATCC 10231 were the most susceptible strains, with MIC values of 3.36 μg/μL.

### 2.4. Inhibition of Biofilm Formation

The effects of strain-specific sub-MICs of the *M. melissophyllum* extract on biofilm biomass and planktonic growth are presented in Table 4. The extract produced a concentration and strain-dependent reduction in biofilm biomass, with the highest effects generally observed at 1/2 MIC. At this concentration, biofilm reduction ranged from 8.95 ± 0.88% for *P. aeruginosa* ATCC 27853 to 16.70 ± 0.45% for *E. coli* ATCC 25922. *C. albicans* ATCC 10231 and *E. coli* ATCC 25922 were the most responsive microorganisms, while *P. aeruginosa* ATCC 27853 showed the lowest reduction in biofilm biomass. At 1/4 MIC, the effect decreased for most strains; however, a clear reduction in biofilm biomass was still observed for *C. albicans* ATCC 10231. At 1/2 MIC and 1/4 MIC, biofilm reduction consistently exceeded the corresponding inhibition of planktonic growth. Planktonic growth inhibition remained below 5.20% at 1/2 MIC and below 2.25% at 1/4 MIC. At 1/8 MIC, both biofilm reduction and planktonic growth inhibition were minimal for all tested microorganisms.

### 2.5. Cytotoxicity Assays

The cytotoxic effect of the *M. melissophyllum* hydroalcoholic extract was evaluated via the CCK-8 assay on three cell lines, namely Ovcar-3, HCT-116 and BJ, following 24 h exposure to five graded concentrations (10.74–53.7 µg/µL).

**Figure 1:**
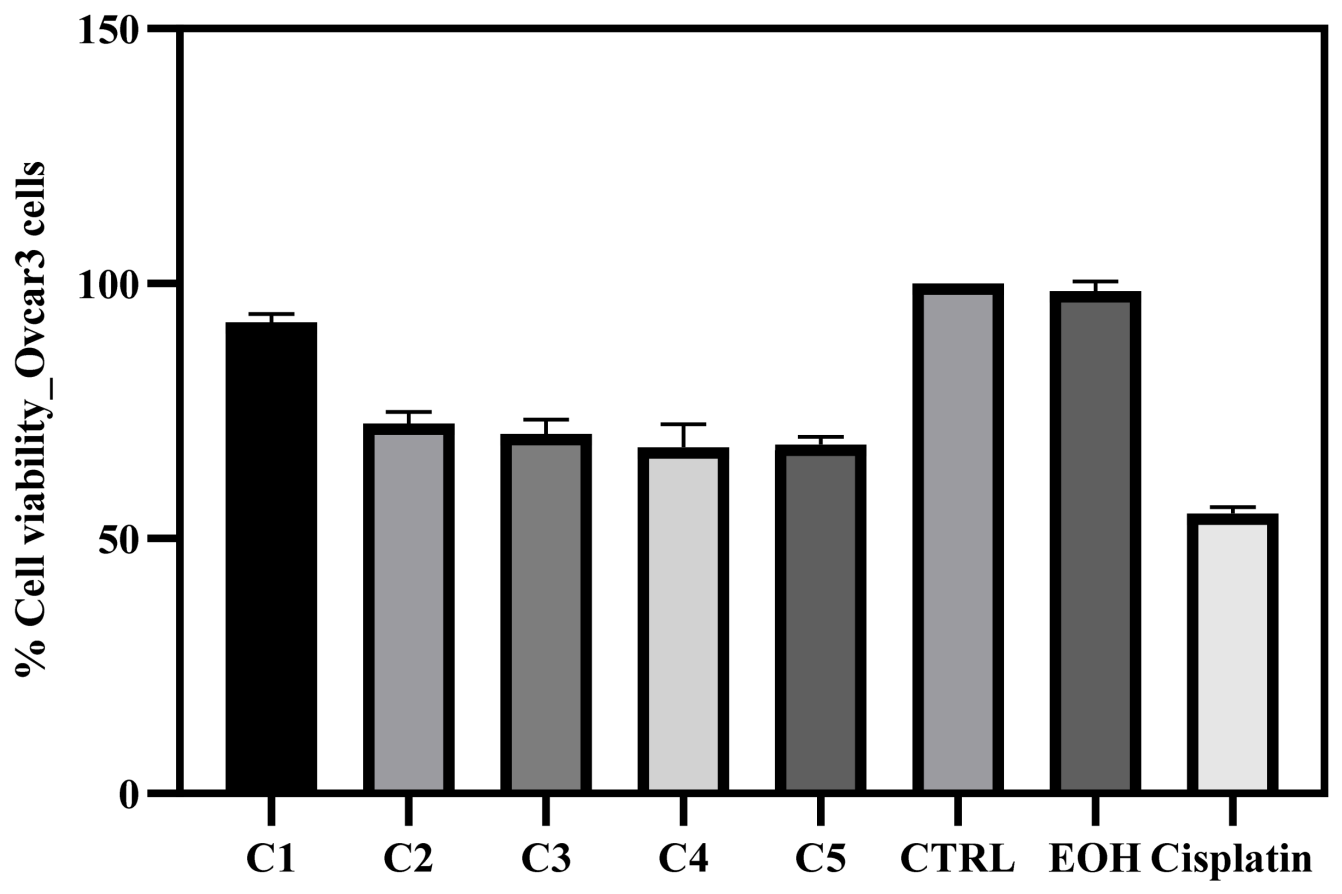
Viability percentages obtained for Ovcar-3 cells after 24 h incubation with *M. melissophyllum* extract. The extract concentrations were ranging from 10.74 to 53.7 µg/µL (Statistical analysis revealed a highly significant overall effect, as determined by one-way ANOVA (F (7,16) = 153.5, *p* < 0.0001, R^2^ = 0.9853). The assumption of homogeneity of variance was satisfied, as confirmed by the Brown–Forsythe test (*p* = 0.5934), indicating no significant differences in variance among groups. Data are presented as the mean ± standard deviation (SD) of three technical replicates (SD for C1 = 1.67, for C2 = 2.26, for C3 = 2.75, for C4 = 4.55, for C5 = 1.46, for Ctrl = 0, for EOH = 1.78, for cisplatin = 1.18).

A differential response was observed between tumor and non-tumor cells. In Ovcar-3 cells, a concentration-dependent decline in viability was evident, ranging from 92.43 ± 1.37% at the lowest concentration (C1) to approximately 68% at the highest doses (C4 and C5), indicating moderate cytotoxic potential (Figure 1).

**Figure 2:**
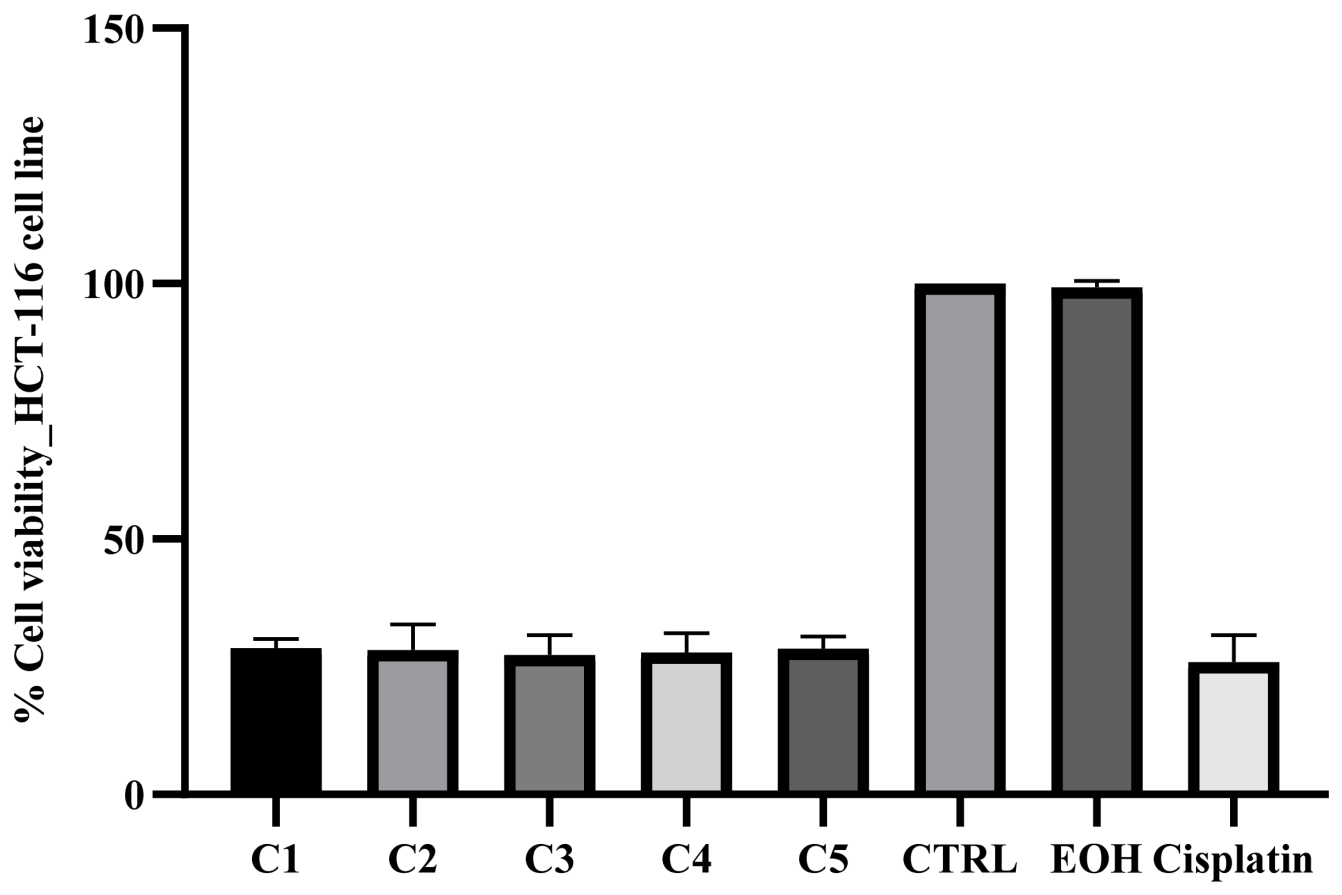
Viability percentages obtained for HCT-116 cells after 24 h incubation with *M. melissophyllum* extract. The extract concentrations were ranging from 10.74 to 53.7 µg/µL. One-way ANOVA showed significant treatment effects on HCT-116 viability (F (7,16) = 284.0, *p* < 0.0001, R^2^ = 0.9920). Homogeneity of variances was confirmed using the Brown–Forsythe test (*p* = 0.7404, ns). Data are presented as the mean ± standard deviation (SD) of three technical replicates (SD for C1 = 1.82, for C2 = 5.00, for C3 = 3.99, for C4 = 3.72, for C5 = 2.31, for Ctrl = 0, for EOH = 1.22, for cisplatin = 5.35).

Conversely, HCT-116 cells demonstrated potent and consistent cytotoxicity across all tested concentrations. Even the lowest dose (C1) resulted in a marked decrease in viability (28.66 ± 1.39%), with minimal variation at higher concentrations (all remaining below 30%), suggesting rapid onset of maximal cytotoxic effect or a plateau response (Figure 2).

**Figure 3:**
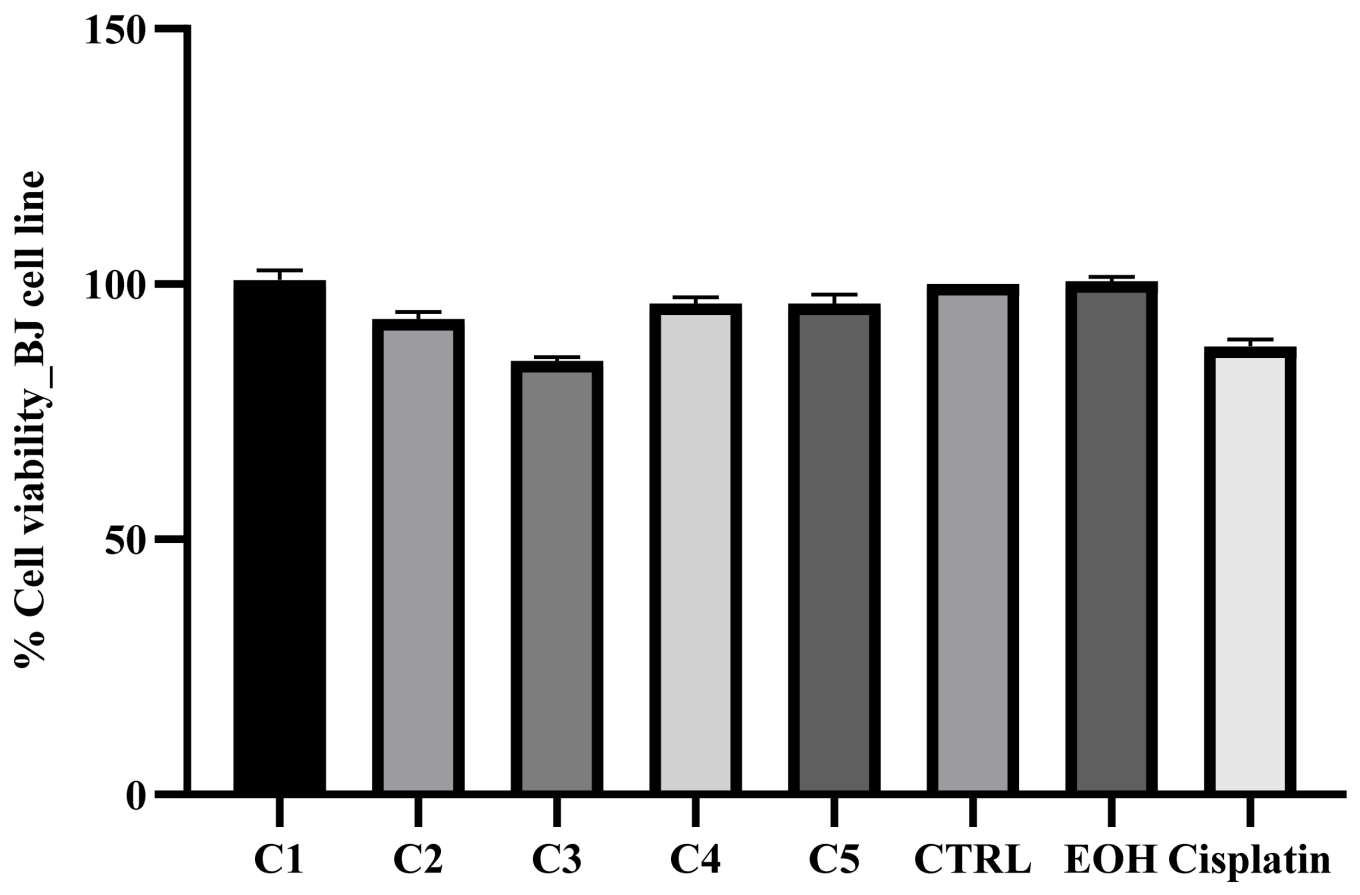
Viability percentages obtained for BJ cells after 24 h incubation with *M. melissophyllum* extract. The extract concentrations were ranging from 10.74 to 53.7 µg/µL. One-way ANOVA showed statistically significant differences in BJ cell viability across treatments (F (7,16) = 66.38, *p* < 0.0001, R^2^ = 0.9667), although all values remained within a non-cytotoxic range. Variance homogeneity was confirmed by the Brown–Forsythe test (*p* = 0.7172, ns). Data are presented as the mean ± standard deviation (SD) of three technical replicates (SD for C1 = 1.86, for C2 = 1.28, for C3 = 0.67, for C4 = 1.19, for C5 = 1.69, for Ctrl = 0, for EOH = 0.87, for cisplatin = 1.45).

In contrast, non-tumor BJ fibroblasts exhibited high tolerance to the extract, maintaining viability above 84% across all concentrations, with the lowest dose slightly exceeding baseline levels (100.79 ± 1.86%) (Figure 3).

In all the performed assays, the vehicle control (50% ethanol in water, *v*/*v*) did not significantly affect cell viability, confirming the specificity of the observed effects. Cisplatin served as a positive control, consistently inducing expected reductions in cell viability across all cell lines.

## 3. Discussions

The amount of phenolic metabolites in extracts plays an important role in their antioxidative behavior. The results obtained in the present study for the quantification of polyphenols, flavonoids and caffeic acid derivatives could be linked with the results obtained for the assessment of their antioxidant capacity. Regarding TPC, TFC and CADC, the obtained results hereby were similar to the ones obtained in previously performed studies [6,17,18]. Antioxidant capacity assays (e.g., FRAP) indicate high activity in *M. melissophyllum*, which is also consistent with previous studies [9,17,18]. Antioxidant activity is demonstrated in vitro in multiple extracts and is supported by in vivo measurements of antioxidant enzyme systems in liver and blood matrices; these activities are consistent with the plant’s phenolic and flavonoid richness and support its therapeutic potential as a natural antioxidant source [18].

LC-MS analysis provided important information related to individual polyphenolic components. Thus, the present study provides the first phytochemical characterization of the polyphenolic profile of *M. melissophyllum* collected from Romania, and the results are consistent with previously published data for samples collected from other European areas but also with some particularities. Overall, our findings confirm that the species is a rich source of phenolic acids, flavonoids in free or glycosylated form, while presenting notable quantitative differences that are related to environmental conditions, plant organs and developmental stage.

The phenolic profiles analyzed by LC-MS method for the Romanian sample is generally aligned with the main compounds previously described in the literature, such as caffeic acid, chlorogenic acid, trans-p-coumaric acid, ferulic acid, luteolin-7-*O*-glucoside. However, the concentrations of these compounds differ from the values reported in other studies from Central and Western Europe [4]. In addition, the extract from the aerial parts of the species also presented previously unreported compounds such as some phenolic compounds such as rosmarinic acid, salicylic acid, carnosol, as well as flavonoid compounds such as apigenin, chrysin, hesperetin, hyperoside, kaempferol, luteolin, naringenin, quercetin, and rutin. Scientific literature reports show regional variability for the Mediterranean and Balkan populations of *M. melissophyllum*, suggesting that phenolic plasticity is a key ecological trait of the species. Also, qualitative and quantitative differences were highlighted by different studies to be due to the ecological factors, including altitude, soil composition, humidity or exposure to sunlight, but also to the harvesting period and the plant material used (the entire flowering aerial part). The variation in the content of polyphenolic compounds, in direct relation to the vegetative mass, with the abundance of flowering is also linked to the exposure to photosynthetically active solar radiation. Exposure of the plant to different levels of shade revealed a significant increase in green mass and abundance of flowering in 30% shade [6]. Added to this are the method of preparation of the extract and the nature and concentration of the solvent used. Seasonal variation also seems to play an important role. According to previous reports, spring samples are richer in coumarin or chlorogenic acid, while summer flowering samples tend to accumulate higher concentrations of flavonoid glycosides such as luteolin-7-*O*-glucoside [4]. This pattern supports the hypothesis that *M. melissophyllum* modulates its metabolic pathways according to phenological stage: early-season compounds are often associated with defense against herbivory and UV stress, while late-season flavonoids may be linked to pollinator attraction or enhanced antioxidant capacity. Differences in the chemical composition of different vegetative organs have been highlighted in previous studies. Our analyzed plant material was a mixture of different proportions of leaves, flowers and stems with a contribution of compounds specific to each vegetative organ, to which are added differences related to microhabitat that also influence the biosynthesis of compounds. Taken together, these findings highlight the fact that *M. melissophyllum* from Romania possesses a comparable phytochemical profile compared to European populations, with some qualitative and quantitative elements of phytochemical differentiation. Thus, the study highlights high levels of chlorogenic acid, trans-p-coumaric acid, caffeic acid, salicylic acid among phenolic acids or hyperoside, luteolin-7-*O*-glucoside luteolin as phenolic components. This polyphenolic profile suggests a promising potential for pharmacological or nutraceutical applications that exploit antioxidant, antimicrobial or antitumor properties.

The IC_50_ value was estimated to be >53.7 µg/μL, as cell viability remained above 50% at all tested concentrations. As 50% inhibition was not experimentally reached within the tested concentration range, this value represents an extrapolated estimate and should be interpreted with caution. Statistical analysis of Ovcar-3 cell viability following treatment with the *M. melissophyllum* extract revealed significant differences among the experimental groups. One-way ANOVA demonstrated a highly significant effect of treatment concentration on cell viability (F (7,16) = 153.5, *p* < 0.0001, R^2^ = 0.9853), indicating that at least one treatment group differed significantly from the others. The assumption of homogeneity of variances was confirmed by the Brown–Forsythe test (F (7,16) = 0.8077, *p* = 0.5934), supporting the use of standard one-way ANOVA. Cisplatin (25 μM) was included as a positive control and reduced cell viability to 25.89 ± 4.83%. As only a single concentration was evaluated, this result serves as a reference for assay performance rather than a direct comparison of cytotoxic potency with the tested extract. The IC_50_ for HCT-116 cells was estimated to be <10.74 µg/μL, as all tested concentrations resulted in cell viabilities below 50%. Treatment of HCT-116 colorectal carcinoma cells with *M. melissophyllum* hydroalcoholic extract resulted in statistically significant differences in cell viability across the tested concentrations. One-way ANOVA revealed a highly significant effect (F (7,16) = 284.0, *p* < 0.0001, R^2^ = 0.9920), indicating that the treatment explained over 99% of the variance in the dataset. Variance homogeneity was assessed using the Brown–Forsythe test, which showed no significant differences in standard deviations among groups (F (7,16) = 0.6093, *p* = 0.7404, ns), validating the use of parametric ANOVA. These results confirm a robust and consistent cytotoxic effect of the extract across the tested concentrations on HCT-116 cells. Minimal viability reductions at higher doses remained within a biologically acceptable range, suggesting that the extract exhibits greater cytotoxic activity toward the tested tumor cells than toward BJ fibroblasts under the experimental conditions. The impact of *M. melissophyllum* hydroalcoholic extract on the viability of non-tumoral BJ fibroblast cells was assessed using one-way ANOVA. Although cell viability remained relatively high across all concentrations, the analysis revealed statistically significant differences among treatment groups (F (7,16) = 66.38, *p* < 0.0001, R^2^ = 0.9667), indicating that the extract induced subtle but measurable changes in viability. The assumption of equal variances was confirmed via the Brown–Forsythe test (F (7,16) = 0.6400, *p* = 0.7172, ns), validating the application of parametric ANOVA. Despite statistical significance, the biological relevance of these changes appears limited, as all viability values remained above 84%, suggesting good tolerability of the extract in normal cells. A limitation of the present study is the concentration range selected for the cytotoxicity assays. For the OVCAR-3 cell line, 50% inhibition of cell viability was not reached within the tested concentrations, whereas for the HCT-116 cell line, the estimated IC_50_ was close to the lower limit of the tested range. Consequently, these IC_50_ values should be interpreted with caution. Future studies using a broader concentration range are needed to enable a more accurate determination of IC_50_ values and to further assess the cytotoxic potency of the extracts.

The antimicrobial activity observed for the *M. melissophyllum* extract may be largely explained by its phenolic composition, characterized by high concentrations of chlorogenic acid, luteolin-7-*O*-glucoside, kaempferol, trans-p-coumaric acid, hyperoside, caffeic acid, salicylic acid, luteolin, and apigenin. The predominance of these compounds suggests that the antimicrobial activity of the extract results from the combined action of flavanols, flavones, and hydroxycinnamic acids acting through multiple complementary mechanisms [20,21]. The net inhibition zones produced by the extract were smaller than those obtained with the corresponding positive controls, indicating limited and strain-dependent diffusible antimicrobial activity. However, because agar-diffusion results are influenced by the solubility and diffusion of individual extract constituents through the agar matrix, the broth microdilution assay provides a more appropriate quantitative assessment of microbial growth inhibition.

Among all identified constituents, kaempferol was the most abundant free flavonoid aglycone compound in the analyzed extract, accounting for a substantial proportion of the quantified phenolic fraction. Kaempferol has been extensively investigated for its antibacterial activity against both Gram-positive and Gram-negative microorganisms. Reported mechanisms include disruption of cytoplasmic membrane function, inhibition of nucleic acid synthesis, interference with bacterial topoisomerases, suppression of biofilm development, and attenuation of virulence-associated pathways. This flavonol showed pronounced activity against *Staphylococcus aureus*, including antibiotic-resistant strains, suggesting that it may represent one of the major contributors to the inhibitory activity observed against both *S. aureus* strains [22,23].

The lower activity observed against *S. aureus* MRSA (ZOI = 1.5 ± 0.15 mm; MIC = 0.460 µg/µL), compared with the methicillin-sensitive *S. aureus* strain, may be explained by the well-known resistance mechanisms of MRSA, these include the presence of PBP2a, the activation of efflux pumps, and changes in the bacterial cell wall that reduce the accumulation of antimicrobial compounds inside the cell [24,25].

The relatively high susceptibility often reported for Gram-positive bacteria, such as *Bacillus cereus* and *S. aureus*, compared with Gram-negative microorganisms, may be partially explained by the structural characteristics of the bacterial cell envelope. Gram-positive bacteria lack the outer membrane that acts as a permeability barrier in Gram-negative species, facilitating the interaction of phenolic compounds with intracellular targets [26].

The activity recorded against *S. enteritidis* and *E. coli* may reflect the capacity of luteolin, apigenin, and caffeic acid to partially compromise outer membrane integrity in enteric pathogens and interfere with virulence-associated gene regulation [20,21,26].

Luteolin and apigenin were also present in considerable amounts in the analyzed extract. These aglycone flavones are among the most extensively studied plant-derived antimicrobials and have demonstrated activity against *Staphylococcus aureus*, *Enterococcus faecalis*, *Escherichia coli*, and *Pseudomonas aeruginosa*. Their antimicrobial mechanisms involve the inhibition of nucleic acid synthesis, interference with membrane-associated enzymes, the suppression of quorum sensing pathways, and the inhibition of biofilm formation. Importantly, both compounds have been reported to potentiate the activity of conventional antibiotics and reduce the virulence of resistant bacterial strains [27,28].

Luteolin-7-*O*-glucoside and hyperoside, the predominant compounds of the extract, may contribute to the antimicrobial activity of *M. melissophyllum* extract. Although glycosylated flavonoids are generally less active than aglycones due to reduced membrane permeability, they may act as precursors of bioactive metabolites. However, glycosylated flavonoids can undergo enzymatic hydrolysis in biological environments, releasing the active aglycone luteolin, which exhibits well-documented bacteriostatic and bactericidal properties against both Gram-positive and Gram-negative pathogens [28,29].

The second most abundant group of antimicrobial constituents consisted of hydroxycinnamic acids, particularly trans-p-coumaric acid and caffeic acid. Trans-p-coumaric acid has been reported to inhibit microbial growth through membrane destabilization, the disruption of proton gradients, and interference with energy metabolism [30,31]. Previous studies have shown that *p*-coumaric acid exhibits particularly strong activity against Gram-positive bacteria and may significantly reduce biofilm formation [30]. Likewise, caffeic acid has been associated with membrane damage, leakage of intracellular constituents, and the inhibition of bacterial adhesion [32]. The coexistence of these two structurally related phenolic acids may result in additive effects that enhance overall antimicrobial efficacy.

Salicylic acid, may further contribute to antimicrobial activity through the inhibition of microbial adhesion and biofilm development. Although generally considered less potent than flavonoids in terms of direct bactericidal activity, salicylic acid has been shown to interfere with quorum sensing and surface colonization processes, thereby limiting the establishment of persistent infections [31].

Rosmarinic acid, despite being detected at a relatively modest concentration, possesses well-documented antibacterial and antibiofilm properties and may contribute disproportionately to biological activity because of its high intrinsic potency [33]. Similar considerations apply to carnosol, which is regarded as one of the most potent antimicrobial diterpenes found in aromatic medicinal plants and exhibits activity against *Staphylococcus aureus*, including MRSA strains [34].

The activity observed against *Candida albicans* may also be explained by the combined action of the identified phytochemicals. Kaempferol, luteolin, caffeic acid, ferulic acid, rosmarinic acid, and apigenin have all been reported to interfere with fungal membrane integrity, ergosterol biosynthesis (leading to increased membrane permeability and subsequent cellular damage), and oxidative homeostasis [33,35,36]. Consequently, the antifungal effect of the extract is likely the result of multiple overlapping mechanisms targeting fungal cell viability and virulence.

Overall, the phytochemical profile suggests that kaempferol is the principal contributor to the antimicrobial activity of the investigated *M. melissophyllum* extract, while trans-p-coumaric acid, hyperoside, caffeic acid, luteolin, apigenin, and salicylic acid provide substantial complementary activity. Rosmarinic acid and carnosol, although present at lower concentrations, may enhance efficacy through antibiofilm and synergistic mechanisms.

The MIC values of the *M. melissophyllum* extract ranged from 3.36 to 13.43 μg/μL, demonstrating a clear strain-dependent response. *Bacillus cereus* ATCC 11778, methicillin-susceptible *Staphylococcus aureus* ATCC 25923, and *Candida albicans* ATCC 10231 were the most susceptible microorganisms, whereas MRSA ATCC 43300 and *Pseudomonas aeruginosa* ATCC 27853 showed the highest MIC values. The fourfold difference between the methicillin-susceptible and methicillin-resistant *S. aureus* strains further indicates that susceptibility may vary substantially within the same species.

Previous evidence for *M. melissophyllum* is limited but similarly suggests selective antimicrobial activity. The ethyl acetate extract was active against *S. enteritidis*, *P. aeruginosa*, and *S. aureus*, whereas no activity was reported against the other tested microorganisms [37]. Considerable variability has also been reported for *Melissa officinalis*. Its total ethanolic extract showed MIC values of 28.80 µg/mL against *P. aeruginosa* and 25.60 µg/mL against *C. albicans*, while the corresponding solvent fractions displayed markedly different activities depending on their composition [38]. In a disk-diffusion study, an ethanolic *M. officinalis* extract tested at 50 mg/mL inhibited *S. aureus*, *E. coli*, *P. aeruginosa*, and *C. albicans*, again indicating microorganism- and extract-dependent activity [39].

Similar variation has been observed within the genus *Mentha*. Ethanolic extracts of nine *Mentha* species generally showed stronger antibacterial activity than extracts obtained with less polar solvents, although the magnitude of inhibition varied among species and bacterial strains [40]. For *Mentha piperita*, a hydroalcoholic extract required substantially higher concentrations to inhibit *Candida* isolates, with complete inhibition generally observed at 62.5 mg/mL [41]. The MIC of 3.36 μg/μL obtained for *C. albicans* in the present study was therefore numerically lower, although direct comparisons remain limited by differences in plant species, extract preparation, microbial strains, and assay design. Overall, the available evidence confirms that the antimicrobial activity of Lamiaceae extracts is strongly influenced by extract composition, solvent, concentration, and target microorganism [42].

The ability of microorganisms to form biofilms is recognized as one of the major virulence factors contributing to chronic and recurrent infections. Cells embedded within biofilms exhibit significantly increased tolerance to antimicrobial agents and host immune responses compared with their planktonic counterparts. In the present study, the antibiofilm activity of the *M. melissophyllum* extract was evaluated at strain-specific sub-MICs, together with planktonic growth, to determine whether the reduction in biofilm biomass could be explained solely by growth inhibition. The effect was concentration-dependent, with the greatest reductions observed at 1/2 MIC and progressively weaker effects at 1/4 and 1/8 MIC. At all tested concentrations, biofilm biomass reduction exceeded the corresponding inhibition of planktonic growth. For example, at 1/2 MIC, *E. coli* ATCC 25922 showed a 16.70% reduction in biofilm biomass, compared with 5.20% inhibition of planktonic growth, indicating that the decrease in crystal violet staining was not attributable only to impaired microbial growth.

The strongest responses at 1/2 MIC were observed for *E. coli* ATCC 25922 and *C. albicans* ATCC 10231, with biofilm biomass reductions of 16.70% and 15.40%, respectively. Lower effects were recorded for *S. aureus* ATCC 25923, MRSA ATCC 43300, and *P. aeruginosa* ATCC 27853. Overall, however, the reductions were modest and should be interpreted as limited, concentration-dependent in the case of antibiofilm activity.

Antibiofilm and anti-adhesion effects have also been reported for related Lamiaceae species, although the magnitude of the response varied according to the extract type, microorganism, and stage of biofilm development. *Mentha piperita* preparations reduced microbial adhesion or biofilm formation in some experimental models, whereas other studies found antimicrobial activity without a corresponding reduction in adhesion [43,44]. Similarly, antibiofilm activity has been reported for *Melissa officinalis*, particularly for its essential oil against bacterial and *Candida* biofilms [45,46,47]. However, most of these studies evaluated essential oils or differently prepared extracts; therefore, direct quantitative comparison with the crude hydroethanolic extract investigated here is limited.

The observed activity may be partly associated with the flavonoids and hydroxycinnamic acids identified in the extract, including luteolin-7-*O*-glucoside, kaempferol, hyperoside, luteolin, apigenin, caffeic acid, *p*-coumaric acid, and rosmarinic acid. Several of these compounds have been reported to interfere with microbial adhesion, quorum sensing, extracellular matrix production, and biofilm maturation, including in *C. albicans* and quorum-sensing-regulated pathways of *P. aeruginosa* [13,33,48]. Given the relatively low magnitude of the effects, the reduction in biofilm biomass is more likely to reflect the combined action of multiple extract constituents than a single dominant mechanism. Because the crystal violet assay measures total attached biomass, it does not distinguish viable from non-viable cells or identify the specific stage of biofilm development affected. Further studies using complementary mechanistic assays are therefore required.

## 4. Materials and Methods

### 4.1. Plant Material

The upper flowering aerial parts of *M. melissophyllum* were harvested at the flowering stage ed from the Hoia-Baciu Forest, Cluj-Napoca (46°46′26″ N 23°31′19″ E), Romania, in June 2024. The vegetal product was dried in shade in the laboratory. The species was identified in the Department of Pharmaceutical Botany of the “Iuliu Hațieganu” University of Medicine and Pharmacy (Cluj-Napoca, Romania) and received the voucher number 122.11.1.1 [49].

### 4.2. Chemical Agents and Apparatus

Chemical agents were purchased from several chemical companies. Aluminum chloride, the Arnow and Folin–Ciocâlteu reagents, ethanol, methanol, sodium acetate, hydrochloric and acetic acids, DPPH, acetonitrile, and ammonium acetate were purchased from MercK KGaA (Darmstadt, Germany). Ferric chloride III, hydroxide and sodium carbonate were purchased from Alfa Aesar (USA/Germany), and 6-hydroxy-2,5,7,8-tetramethylchroman-2-carboxylic acid (Trolox) and 2,4,6-tripyridyl-s-triazine (TPTZ) were purchased from Fluka Chemie GmbH (Steinheim, Germany). All references used in the LS-MS analysis were obtained from Phytolab (Vestenbergsgreuth, Germany). The spectrophotometer was Agilent Cary 60 UV-vis (Santa Clara, CA, USA). The Ovcar (HTB-161), HCT116 (CCL-247), BJ-CRL 2522 cell lines used in this study were ATCC-derived and were kindly provided by the “Ion Chiricuță” Oncology Institute in Cluj-Napoca, Romania. The reagents used for the cytotoxicity and antimicrobial assays included RPMI-1640 medium (Gibco Life Technologies, Paisley, UK), fetal bovine serum (FBS) and L-glutamine (Sigma-Aldrich, St. Louis, MO, USA), gentamicin and fluconazole (Oxoid Ltd., Hampshire, UK), the McFarland standard (bioMérieux, Marcy-l’Étoile, France), and Mueller–Hinton (MH) agar and Sabouraud dextrose agar (Merck, Darmstadt, Germany).

### 4.3. Extraction Technique

The dried upper aerial parts of *M. melissophyllum* were ground using a Grindomix knife mill GM 200 (Retsch GmbH, Haan, Germany) and then extracted at 60 °C, for 30 min, on a water bath, using 10.00 g of vegetal material and 100 mL of 50% ethanol (50% ethanol in water *v*/*v*). Then, the extract was filtered and made up to 100 mL with solvent before being centrifuged, and the clear solution was collected and stored in the refrigerator until used for chemical and biological analysis [49,50,51,52]. The extraction yield was 26.85% (g/g) and the dry residue concentration was 5.37% (g/mL). The obtained extract was used for biological assays and results were reported per dry extract (μg/μL).

### 4.4. Spectrophotometric Analysis

#### 4.4.1. Total Polyphenolic Content (TPC) Method

Using the Folin–Ciocalteu reagent and a sodium carbonate solution (29%), the total polyphenolic content (TPC) in the *M. melissophyllum* extract was spectrophotometrically determined. Absorbance of the solution was measured at 760 nm and using a gallic acid calibration curve (R^2^ = 0.998), the results were expressed in mg gallic acid equivalents (GAE)/mL *M. melissophyllum* extract [49,50,53].

#### 4.4.2. Total Flavonoid Content (Tfc) Method

The flavonoid concentration of the *M. melissophyllum* sample was determined after complexation with aluminum chloride (AlCl_3_) and the absorbance of the yellow solution was measured at 430 nm. The results representing the average of three determinations were expressed in mg rutin equivalents (RE)/mL *M. melissophyllum* extract after establishing a rutin calibration curve (R^2^ = 0.998) [49,50,53].

#### 4.4.3. Caffeic Acid Derivative Content (CADC) Method

The content of caffeic acid derivatives (CADC) in the extract of *M. melissophyllum* was determined using a spectrophotometer, using the Arnow reagent based on a standard curve generated with caffeic acid (R^2^ = 0.989) at 500 nm. The CAD content in the sample was expressed in mg of caffeic acid equivalents (CAE)/mL *M. melissophyllum* extract [49,50,53].

### 4.5. LC–MS Analysis

LC–MS analyses were carried out using a Shimadzu Nexera I LC–MS-8045 UHPLC system (Kyoto, Japan) equipped with a quaternary pump, an autosampler, an electrospray ionization (ESI) source, and a quadrupole rod mass spectrometer. Compound separation was achieved on a Luna C18 reversed-phase column (150 mm × 4.6 mm × 3 mm, 100 Å, Phenomenex, Torrance, CA, USA). The column temperature was maintained at 40 °C degrees. The mobile phase consisted of a gradient mixture of analytical-grade methanol, ultrapure water and formic acid as an organic modifier (Table 5). The used flow rate was maintained at 0.5 mL/min and the total analysis time was 35 min. To carry out the analysis, the extract was diluted 1:5 *v*/*v* with methanol of analytical grade. The injection volume (both for references and samples) was 10 μL. Mass spectrometric detection was performed in both positive and negative ESI modes under multiple reaction monitoring (MRM) conditions. The interface temperature was set at 300 °C. Nitrogen (35 psi, 10 L/min) was used for vaporization and as drying gas. The capillary potential was set at +3000 V. The identification of compounds was based on comparisons of retention times, MS spectra, and MRM transitions with those of references. Quantification relied on the primary transition for each analyte. Calibration curves with excellent linearity (R^2^ = 0.9964–0.9999) were established for all compounds and reference standards (Table 6 and Table 7). Results were expressed as μg/g dw vegetal product. Each sample was analyzed in triplicate [50,54].

### 4.6. Antioxidant Capacity Methods

The DPPH radical scavenging technique and the ferric ion reducing (FRAP) assay were used to evaluate the antioxidant potential of the *M. melissophyllum* sample.

#### 4.6.1. DPPH Method

The antioxidant activity is based on the property of polyphenolic compounds in the plant extract to donate a hydrogen proton to the DPPH• radical (violet) and obtain a yellow color. The following represents a brief description of the procedure: A total of 2 mL of ethanolic DPPH solution (0.1 g/L) was mixed with 2 mL of *M. melissophyllum* extract at different concentrations (31.25–250 μL). After 30 min of reaction, the absorbance of the sample (As; DPPH radical + sample) and the control (Ac; DPPH radical + ethanol) were measured at 517 nm, and then the IC_50_ value (mg/mL) was calculated. The standard antioxidant used was Trolox, and the test was performed in triplicate [49,50,55].

#### 4.6.2. FRAP Test

The antioxidant potential of the *M. melissophyllum* extract was determined using the FRAP test based on the reducing power of the polyphenols in the sample to reduce the ferric ion to the ferrous ion, in the form of a blue complex (Fe^2+^/TPTZ: 2,4,6-tripyridyl-s-triazine). The absorbance of the sample was measured at 450 nm. The standard curve was prepared with different concentrations of the antioxidant standard Trolox (R^2^ = 0.995). The FRAP results were expressed in mg TE/mL *M. melissophyllum* extract [49,50,55].

### 4.7. Antimicrobial Activity and MIC

The antimicrobial efficacy of *M. melissophyllum* extract was assessed against the following indicator bacteria: *Bacillus cereus* ATCC 11778, *Staphylococcus aureus* ATCC 25923, *Staphylococcus aureus* MRSA ATCC 43300, *Escherichia coli* ATCC 25922, *Salmonella enteritidis* ATCC 25928, *Pseudomonas aeruginosa* ATCC 27853, and *Candida albicans* ATCC 10231 (ATCC, American Type Culture Collection, Manassas, VA, USA). The pathogenic strains were aerobically grown in 10 mL BHI broth at 37 °C for 18 h; bacterial suspensions were adjusted to a concentration of 0.5 McFarland to give a cell density of ~10^8^ CFU/mL.

#### 4.7.1. Agar Well-Diffusion Assay

The antimicrobial activity of the *M. melissophyllum* extract was evaluated according to Matuschek et al., with minor modifications [56]. Briefly, 15 mL of nutrient agar was inoculated with a 0.5 McFarland suspension of the test pathogens and poured into Petri dishes (Ø 90 mm). Wells (0.6 mm diameter) were then prepared in the solidified agar, and 50 µL of the extract was added to each well. As a negative control, 50 µL of 50% ethanol was used, while vancomycin (30 µg) served as the positive control for Gram-positive bacteria, gentamicin (10 µg) for Gram-negative bacteria, and fluconazole (25 µg) for yeasts. After incubation at 37 °C for 24–48 h, the total diameter of the clear inhibition zone was measured. The results were expressed as the net zone of inhibition (ZOI), calculated by subtracting the 6 mm well diameter from the total diameter of the clear zone. All assays were performed in triplicate.

#### 4.7.2. Determination of the Minimum Inhibitory Concentrations (Microplate Assay)

The minimum inhibitory concentration (MIC) was defined as the lowest concentration of antimicrobial agent that completely inhibits the growth of a microorganism. MIC values for the *M. melissophyllum* extracts were determined using the microdilution method, according to the Clinical and Laboratory Standards Institute (CLSI) guidelines M07-A9 [57] with minor modifications. For this assay, bacterial strains were cultured in nutrient broth (Merck, Darmstadt, Germany, 105443), while fungal strains were grown in Sabouraud Dextrose Broth (SDB). Pathogenic strains were initially incubated in 10 mL BHI broth at 37 °C for 18 h. The bacterial inoculum was adjusted to 0.5 McFarland standard (approximately 10^8^ CFU/mL) and further diluted to a final concentration of 5 × 10^5^ CFU/mL per well. The fungal inoculum was prepared at 10^6^ CFU/mL using the same standardization approach. The assay was performed in 96-well microplates, with cultures incubated at 37 °C for 24 h. *M. melissophyllum* extract was tested at concentrations of 50%, 25%, 12.5%, 6.25%, 3.12%, and 1.56% (*v*/*v*) corresponding to the dry extract concentrations of 26.85, 13.43, 6.71, 3.36, 1.68, and 0.84 μg/μL, respectively, based on a dry-residue concentration of 53.7 μg/μL, with a final well volume of 200 µL and 10 µL of microbial inoculum per well.

Positive growth controls (medium with inoculated microorganisms, without extract) and negative controls (extract in sterile culture medium) were included in parallel. In addition, the ethanol control (50% ethanol in water, *v*/*v*) was tested at equivalent dilutions to exclude any antimicrobial effect attributable to the solvent. Plates were incubated under continuous shaking for 24 h at 37 °C using a BioTek Synergy 2 multimode microplate reader (BioTek Instruments, Winooski, VT, USA), and optical density (OD600) was recorded every 15 min. MIC was defined as the lowest extract concentration that resulted in complete inhibition of microbial growth. All assays were performed in three independent experiments.

#### 4.7.3. Inhibition of Biofilm Formation and Planktonic Growth

The effects of the tested extract on biofilm biomass and planktonic growth were evaluated using a microtiter plate assay. The following microorganisms were included: *S. aureus* ATCC 25923, *S. aureus* MRSA ATCC 43300, *P. aeruginosa* ATCC 27853, *E. coli* ATCC 25922, and *C. albicans* ATCC 10231. The extract was tested at strain-specific sub-inhibitory concentrations corresponding to 1/2 MIC, 1/4 MIC, and 1/8 MIC. Because the MIC values differed among the tested microorganisms, the absolute extract concentrations were calculated separately for each strain based on its experimentally determined MIC.

For the planktonic growth assay, 100 µL of a standardized microbial suspension containing 10^6^ CFU/mL was added to each well of sterile 96-well microtiter plates containing using BHI and SDB supplemented with 1% glucose. The appropriate volume of *M. melissophyllum* extract was added to obtain the strain-specific concentrations corresponding to 1/2 MIC, 1/4 MIC, and 1/8 MIC. The final volume was adjusted to 200 µL with Mueller–Hinton broth, resulting in a final microbial inoculum of approximately 5 × 10^5^ CFU/mL per well. The plates were incubated aerobically at 37 °C for 48 h. After incubation, optical density was measured at 600 nm using a BioTek Synergy 2 multimode microplate reader (BioTek Instruments, Winooski, VT, USA). Planktonic growth inhibition was expressed as the percentage decrease in blank-corrected OD_600_ relative to the untreated growth control.

For biofilm formation, the assay was performed in parallel using BHI and SDB supplemented with 1% glucose. The same inoculum density, extract concentrations, final volume, and incubation conditions were used. After incubation, the culture medium and non-adherent cells were removed, and the wells were gently washed three times with sterile phosphate-buffered saline. The plates were air-dried, and the attached biofilm biomass was stained with 100 µL of 1% (*w*/*v*) crystal violet for 30 min at room temperature. Excess stain was removed by washing the wells three times with sterile distilled water. The bound dye was solubilized with absolute ethanol, and absorbance was measured at 570 nm using the same microplate reader. Biofilm biomass reduction was expressed as the percentage decrease in blank-corrected OD_570_ relative to the untreated biofilm control.

Untreated microbial cultures were included as growth and biofilm controls. Solvent controls containing the same final ethanol concentration as the corresponding extract treatments were tested in parallel. Extract blanks containing the corresponding extract concentration in sterile medium, without microbial inoculum, were included to correct for the intrinsic absorbance of the extract and medium. Sterile medium blanks were used to correct the untreated controls. All absorbance values were corrected using the corresponding blanks before calculating planktonic growth inhibition and biofilm biomass reduction. All assays were performed in triplicate, and the results were expressed as mean ± standard deviation [58,59,60].

### 4.8. Cytotoxicity Assays

The cytotoxic activity of the tested extracts was evaluated on three distinct cell lines: normal diploid human foreskin fibroblasts (BJ), and two cancer-derived lines—the human colorectal carcinoma line HCT-116 (CCL-247), and the ovarian adenocarcinoma line Ovcar-3 (HTB-161). BJ fibroblasts were cultured in Minimum Essential Medium (MEM), supplemented with 10% fetal bovine serum (FBS) and 1% antibiotic–antimycotic solution. HCT-116 cells were maintained in McCoy’s 5A medium (Sigma-Aldrich) with the same supplement concentrations, while Ovcar-3 cells were grown in RPMI 1640 medium (Lonza, Basel, Switzerland), enriched with 20% FBS and 1% antibiotic–antimycotic (Gibco, Grand Island, NY, USA). All cell cultures were incubated at 37 °C in a humidified atmosphere containing 5% CO_2_. Cells were seeded at a density of 1 × 10^4^ cells per well in 96-well culture plates and allowed to adhere for 24 h. Following incubation, cultures were treated with five concentrations of *M. melissophyllum* extract ranging from 10.74 to 53.7 μg/μL. Appropriate controls were included in all assays: untreated cells (negative control), vehicle control (50% ethanol in water, *v*/*v*), and a cytotoxicity control consisting of cisplatin at 25 µM (positive control). Cisplatin was included as a positive control to verify the responsiveness of the cell lines. It was used as a reference compound and was not intended for the determination of a concentration–response curve or direct comparison of potency with the tested extracts. Cell viability was assessed using the Cell Counting Kit-8 (CCK-8, Sigma-Aldrich, St. Louis, MO, USA), following the manufacturer’s instructions. After 24 h of extract exposure, CCK-8 reagent was added, and plates were further incubated for 90 min. Absorbance was measured at 450 nm using a BioTek Synergy 2 microplate reader (Winooski, VT, USA). Viability was expressed as a percentage relative to the negative control. All assays were performed in triplicate. The half-maximal inhibitory concentration (IC_50_) values were determined by nonlinear regression analysis of dose–response curves [49,54].

### 4.9. Statistical Analysis

Data are presented as the mean ± standard deviation (SD) of three technical replicates for each experimental condition. Statistical analysis was performed using one-way analysis of variance (ANOVA), followed by Tukey’s multiple comparisons test. Differences were considered statistically significant at *p* < 0.05.

## 5. Conclusions

The present study demonstrated that *M. melissophyllum* is a rich source of polyphenolic compounds, as confirmed by spectrophotometric analyses and LC–MS profiling. The hydroethanolic extract exhibited notable in vitro antioxidant activity, highlighted by its capacity to neutralize the DPPH radicals and reduce the ferric ion in the FRAP method. Furthermore, the extract showed measurable but limited and strain-dependent antimicrobial effects, which were lower than those produced by the corresponding reference antimicrobial agents.

Biological evaluation revealed that the extract was well tolerated by normal human fibroblasts while exerting inhibitory effects on the colorectal (HCT-116) and ovarian (OVCAR-3) cancer cells, suggesting selective cytotoxic potential. These findings support the potential of *M. melissophyllum* as a valuable source of bioactive phytochemicals with antioxidant, antimicrobial, and cytotoxic effects.

Overall, the results provide a scientific basis for the traditional use of *M. melissophyllum* and highlight its potential for further development in pharmaceutical, nutraceutical, and health-promoting applications. Additional studies focusing on the analysis of other classes of active constituents, the elucidation of mechanisms of action, and in vivo validation are warranted to fully explore its therapeutic potential.

## Figures and Tables

**Table 1:** Phenolic, flavonoidic, caffeic acid derivative contents and antioxidant activity of *M. melissophyllum* extract.

Sample	TPC (mg GAE/g)	TFC (mg RE/g)	CADC (mg CAE/g)	DPPH (IC_50_ mg/mL)	FRAP (mg TE/mL)
*M. melissophyllum* extract	18.41 ± 0.13	5.93 ± 0.06	10.96 ± 0.16	0.18 ± 0.01 ^a^	1.060 ± 0.77
Trolox	-	-	-	0.012 ± 0.003	-

Notes: Each value is the mean ± SD of three independent measurements; TPC: total polyphenolic content; TFC: total flavonoid content; CADC: caffeic acid derivative content; GAE, RE, CAE, TE: gallic acid, rutin, caffeic acid, Trolox equivalents, ^a^ *p* < 0.001 (extract vs. Trolox standard).

**Table 2:** Phenolic compounds identified and quantified by LC-MS in the composition of the tested *M. melissophyllum* extract.

Compound	Retention Time (min)		*m*/*z* and Main Transition		Concentration (μg/g dw Vegetal Product)
	Reference	Separated Compound	Reference	Separated Compound	Value
Caffeic acid	13.8	14.2	179.0 > 135.0	179.0 > 135.0	2120 ± 3.51
Chlorogenic acid	12.0	12.5	353.0 > 191.0	353.0 > 191.0	173,550 ± 0.41
trans-p-coumaric acid	17.5	16.6	163.0 > 119.0	163.0 > 119.0	4570 ± 11.5
Ferulic acid	18.4	17.5	193.0 > 134.0	193.0 > 134.0	270 ± 1.2
Rosmarinic acid	21.4	21.8	358.9 > 161.0	358.9 > 161.0	350 ± 2.1
Salicylic acid	23.5	24.2	137.0 > 93.0	137.0 > 93.0	1790 ± 1.9
Apigenin	28.2	28.6	269.0 > 117.0	269.0 > 117.0	1260 ± 2.4
Carnosol	30.6	30.8	329.1 > 285.1	329.1 > 285.1	50 ± 0.1
Chrysin	29.7	30.2	253.0 > 143.0	253.0 > 143.0	240 ± 0.5
Hesperetin	27.1	26.3	301.0 > 164.0	301.0 > 164.0	210 ± 0.5
Hyperoside	20.3	21.1	463.1 > 300.0	463.1 > 300.0	2800 ± 7.1
Kaempferol	27.9	27.4	285.0 > 187.0	285.0 > 187.0	14,610 ± 22.5
Luteolin	26.9	27.4	287.0 > 153.0	287.0 > 153.0	1380 ± 7.2
Luteolin-7-*O*-glucoside	19.9	20.4	447.0 > 284.9	447.0 > 284.9	45,160 ± 105.1
Naringenin	26.3	27.0	271.0 > 119.0	271.0 > 119.0	260 ± 0.5
Quercetin	25.7	26.3	300.9 > 151.0	300.9 > 151.0	530 ± 2.4
Rutin	20.3	20.8	609.0 > 300.0	609.0 > 300.0	290 ± 0.8

Notes: Values represent the mean ± standard deviations of three independent measurements.

**Table 3:** Antimicrobial activity of *M. melissophyllum* ethanolic extracts against the tested pathogenic microorganisms.

Type of Microorganism	Tested Microbial Strains	Positive Control ZOI (mm)	ZOI (mm)	MIC (µg/µL)
Gram-positive strains	*Bacillus cereus* ATCC 11778	19.50 ± 0.10	7.67 ± 0.11	3.36
	*S. aureus* MSSA ATCC 25923	20.0 ± 0.08	6.78 ± 0.11	3.36
	*S. aureus* MRSA ATCC 43300	18.0 ± 0.05	1.5 ± 0.15	13.43
Gram-negative strains	*E. coli* ATCC 25922	19.25 ± 0.20	4.25 ± 0.79	6.71
	*P. aeruginosa* ATCC 27853	15.36 ± 0.25	2.50 ± 0.10	13.43
	*S. enteritidis* ATCC 25928	17.29 ± 0.07	6.25 ± 0.31	6.71
Yeast strain	*C. albicans* ATCC 10231	23.00 ± 0.25	7.14 ± 0.85	3.36

Note: Values are expressed as mean ± SD (n = 3). Positive controls were vancomycin (30 µg) for Gram-positive bacteria, gentamicin (10 µg) for Gram-negative bacteria, and fluconazole (25 µg) for *C. albicans*. ZOI values represent the net zone of inhibition, calculated by subtracting the 6 mm well diameter from the total diameter of the clear zone.

**Table 4:** Effects of strain-specific sub-MICs of the tested extract on biofilm formation and planktonic growth inhibition of different microorganisms.

Microbial Strain	½ MIC		¼ MIC		⅛ MIC	
	Biofilm Reduction (%)	Planktonic Inhibition (%)	Biofilm Reduction (%)	Planktonic Inhibition (%)	Biofilm Reduction (%)	Planktonic Inhibition (%)
*S. aureus* ATCC 25923	12.45 ± 0.20	4.10 ± 0.52	8.38 ± 0.24	1.85 ± 0.31	1.10 ± 0.30	<1
*S. aureus* MRSA ATCC 43300	9.80 ± 0.55	3.90 ± 0.47	4.72 ± 0.31	1.70 ± 0.28	0.65 ± 0.22	<1
*P. aeruginosa* ATCC 27853	8.95 ± 0.88	4.60 ± 0.61	4.15 ± 0.17	1.95 ± 0.34	0.30 ± 0.18	<1
*E. coli* ATCC 25922	16.70 ± 0.45	5.20 ± 0.58	9.25 ± 0.42	2.10 ± 0.37	0.10 ± 0.35	<1
*C. albicans* ATCC 10231	15.40 ± 0.30	4.80 ± 0.49	11.60 ± 0.19	2.25 ± 0.42	2.85 ± 0.32	<1

**Table 5:** Gradient concentration of the LC–MS mobile phase.

Time (min)	Methanol	Water	2% Formic Acid in Water
0.00	5	90	5
3.00	15	70	15
6.00	15	70	15
9.00	21	58	21
13.00	21	58	21
18.00	30	41	29
22.00	30	41	29
26.00	50	0	50
29.00	50	0	50
29.01	5	90	5
35.00	5	90	5

**Table 6:** Identification parameters of the LC-MS analysis for references.

Name of Standard	Retention Time (min)	*m*/*z*, and Main Transition	MRM	Other Transitions
Caffeic acid	13.8	179.0 > 135.0	Negative	179.0 > 134.0 179.0 > 89.0
Chlorogenic acid	11.9	353.0 > 191.0	Negative	
trans-p-coumaric acid	17.5	163.0 > 119.0	Negative	163.0 > 93.0
Ferulic acid	18.4	193.0 > 134.0	Negative	193.0 > 178.0
Rosmarinic acid	21.4	358.9 > 161.0	Negative	358.9 > 133.0
Salicylic acid	23.5	137.0 > 93.0	Negative	137.0 > 75.0 137.0 > 65.0
Apigenina	28.1	269.0 > 117.0	Negative	
Chrysin	29.7	253.0 > 143.0	Negative	253.0 > 119.0 253.0 > 107.0
Hesperetin	27.0	301.0 > 164.0	Negative	301.0 > 136.0 301.0 > 108.0
Hyperoside	20.3	463.1 > 300.0	Negative	463.1 > 301.0
Kaempferol	27.9	285.0 > 187.0	Negative	285.0 > 151.0 285.0 > 133.0
Luteolin	26.8	287.0 > 153.0	Positive	
Luteolin-*7-O*-glucosid	19.9	447.0 > 284.9	Negative	
Naringenin	26.2	271.0 > 119.0	Negative	271.0 > 107.0
Quercetin	25.4	300.9 > 151.0	Negative	300.9 > 121.0
Rutoside	20.2	609.0 > 300.0	Negative	609.0 > 301.0 609.0 > 271.0
Carnosol	30.7	329.1 > 285.1	Negative	

**Table 7:** Quantitative parameters of the LC-MS analysis for references.

Reference	Concentration Range	Calibration Curve Equation	Correlation Factor	LOD	LOQ
Caffeic acid	0.11–1.10	A = 4 × 10^7^ × c − 319,689	0.9998	3.20	4.80
Chlorogenic acid	0.13–1.30	A = 2 × 10^8^ × c − 269,699	0.9997	5.00	8.00
trans-p-coumaric acid	0.16–1.60	A = 3 × 10^7^ × c + 36,967	0.9995	2.50	4.90
Ferulic acid	0.100–1.000	A = 5 × 10^6^ × c − 50,000	0.9992	4.00	6.00
Rosmarinic acid	0.028–0.278	A = 2 × 10^8^ × c − 6664.7	0.9996	0.10	0.20
Salicylic acid	0.16–1.60	A = 4 × 10^7^ × c + 44,120	0.9997	1.50	2.00
Apigenin	0.10–0.98	A = 2 × 10^8^ × c + 15,916	0.9999	0.20	0.30
Chrysin	0.10–1.00	A = 1 × 10^8^ × c − 82,818	0.9997	3.00	5.00
Hesperetin	0.10–1.00	A = 6 × 10^7^ × c − 49,247	0.9974	3.00	5.00
Hyperoside	0.012–0.107	A = 4 × 10^8^ × c − 567,182	0.9986	0.60	0.90
Kaempferol	0.10–1.00	A = 1 × 10^7^ × c − 20,574	0.9996	0.80	1.20
Luteolin	0.01–0.10	A = 2 × 10^8^ × c − 2295.4	0.9977	0.05	0.07
Luteolin-*7-O*-glucoside	0.07–0.70	A = 1 × 10^9^ × c − 700,317	0.9990	3.00	4.00
Naringenin	0.16–1.60	A = 3 × 10^8^ × c – 43,443	0.9999	0.60	0.90
Quercetin	0.09–0.91	A = 5 × 10^7^ × c − 9556	0.9964	0.80	1.10
Carnosol	0.022–0.220	A = 1 × 10^9^ × c − 253,279	0.9997	1.00	2.00

Note: A = Area; c = concentration (mg/mL); Concentration range (mg/mL); LOD = limit of detection (μg/mL); LOQ = limit of quantification (μg/mL).

## Data Availability

The original contributions presented in the study are included in the article; further inquiries can be directed to the corresponding author.

## Supplementary Materials

The following supporting information can be downloaded at https://www.mdpi.com/article/10.3390/antibiotics15080742/s1. Figure S1. LC-MS chromatogram and mass fragmentation pattern obtained for the identification of caffeic acid in the *M. melissophyllum* extract; Figure S2. LC-MS chromatogram and mass fragmentation pattern obtained for the identification of chlorogenic acid in the *M. melissophyllum* extract; Figure S3. LC-MS chromatogram and mass fragmentation pattern obtained for the identification of trans-p-coumaric acid in the *M. melissophyllum* extract; Figure S4. LC-MS chromatogram and mass fragmentation pattern obtained for the identification of ferulic acid in the *M. melissophyllum* extract; Figure S5. LC-MS chromatogram and mass fragmentation pattern obtained for the identification of rosmarinic acid in the *M. melissophyllum* extract; Figure S6. LC-MS chromatogram and mass fragmentation pattern obtained for the identification of salicylic acid in the *M. melissophyllum* extract; Figure S7. LC-MS chromatogram and mass fragmentation pattern obtained for the identification of apigenin in the *M. melissophyllum* extract; Figure S8. LC-MS chromatogram and mass fragmentation pattern obtained for the identification of carnosol in the *M. melissophyllum* extract; Figure S9. LC-MS chromatogram and mass fragmentation pattern obtained for the identification of chrysin in the *M. melissophyllum* extract; Figure S10. LC-MS chromatogram and mass fragmentation pattern obtained for the identification of hesperetin in the *M. melissophyllum* extract; Figure S11. LC-MS chromatogram and mass fragmentation pattern obtained for the identification of hyperoside in the *M. melissophyllum* extract; Figure S12. LC-MS chromatogram and mass fragmentation pattern obtained for the identification of kaempferol in the *M. melissophyllum* extract; Figure S13. LC-MS chromatogram and mass fragmentation pattern obtained for the identification of luteolin in the *M. melissophyllum* extract; Figure S14. LC-MS chromatogram and mass fragmentation pattern obtained for the identification of luteolin-7-*O*-glucoside in the *M. melissophyllum* extract; Figure S15. LC-MS chromatogram and mass fragmentation pattern obtained for the identification of naringenin in the *M. melissophyllum* extract; Figure S16. LC-MS chromatogram and mass fragmentation pattern obtained for the identification of quercetin in the *M. melissophyllum* extract; Figure S17. LC-MS chromatogram and mass fragmentation pattern obtained for the identification of rutoside in the *M. melissophyllum* extract.

## References

[1] Burescu L., Nuțu I., Morar (Burescu) E.A. (2019). Forests Vegetation, Forest Habitats of Community Interest in North-West Romania, Included in the Project “Priority Habitats of Forest Steppe and Hilly Piedmonts”, Potential Threats and Monitoring of Conservation Status (II). Ann. Univ. Oradea.

[2] Radutoiu D. (2020). Ornamental Plants Species from Spontaneous Flora in Oltenia Region, Romania. Sci. Pap. Ser. B Hortic..

[3] Skrzypczak-Pietraszek E., Pietraszek J. (2014). Seasonal Changes of Flavonoid Content in *Melittis melissophyllum* L. (Lamiaceae). Chem. Biodivers..

[4] Szymborska-Sandhu I., Przybył J.L., Pióro-Jabrucka E., Jędrzejuk A., Węglarz Z., Bączek K. (2020). Effect of Shading on Development, Yield and Quality of Bastard Balm Herb (*Melittis melissophyllum* L.). Molecules.

[5] Maggi F., Papa F., Cristalli G., Sagratini G., Vittori S., Giuliani C. (2010). Histochemical Localization of Secretion and Composition of the Essential Oil in *Melittis melissophyllum* L. Subsp. Melissophyllum from Central Italy. Flavour Fragr. J..

[6] Szymborska-Sandhu I. (2020). Developmental and Chemical Characteristics of *Melittis melissophyllum* L. In Limited Access of Sunlight. Herba Pol..

[7] Haas R.A., Crișan I., Vârban D., Vârban R. (2024). Aerobiology of the Family Lamiaceae: Novel Perspectives with Special Reference to Volatiles Emission. Plants.

[8] Skrzypczak-Pietraszek E., Pietraszek J. (2012). Chemical Profile and Seasonal Variation of Phenolic Acid Content in Bastard Balm (*Melittis melissophyllum* L., Lamiaceae). J. Pharm. Biomed. Anal..

[9] Szymborska-Sandhu I., Przybył J.L., Kosakowska O., Bączek K., Węglarz Z. (2020). Chemical Diversity of Bastard Balm (*Melittis Melisophyllum* L.) as Affected by Plant Development. Molecules.

[10] Maggi F., Bílek T., Lucarini D., Papa F., Sagratini G., Vittori S. (2009). *Melittis melissophyllum* L. Subsp. Melissophyllum (Lamiaceae) from Central Italy: A New Source of a Mushroom-like Flavour. Food Chem..

[11] Maggi F., Papa F., Vittori S. (2012). Gas Chromatography for the Characterization of the Mushroom-like Flavor in *Melittis melissophyllum* L. (Lamiaceae). J. Essent. Oil Res..

[12] Kiss A., Papp V.A., Pál A., Prokisch J., Mirani S., Toth B.E., Alshaal T. (2025). Comparative Study on Antioxidant Capacity of Diverse Food Matrices: Applicability, Suitability and Inter-Correlation of Multiple Assays to Assess Polyphenol and Antioxidant Status. Antioxidants.

[13] Davidova S., Galabov A.S., Satchanska G. (2024). Antibacterial, Antifungal, Antiviral Activity, and Mechanisms of Action of Plant Polyphenols. Microorganisms.

[14] Venditti A., Frezza C., Caretti F., Gentili A., Serafini M., Bianco A. (2016). Constituents of Melittis Melissophyllum Subsp. Albida. Nat. Prod. Commun..

[15] Venditti A., Frezza C., Guarcini L., Maggi F., Bianco A., Serafini M. (2016). Reassessment of *Melittis melissophyllum* L. Subsp. Melissophyllum Iridoidic Fraction. Nat. Prod. Res..

[16] Maggi F., Papa F., Cristalli G., Sagratini G., Vittori S. (2010). Characterisation of the Mushroom-like Flavour of *Melittis melissophyllum* L. Subsp. Melissophyllum by Headspace Solid-Phase Microextraction (HS-SPME) Coupled with Gas Chromatography (GC-FID) and Gas Chromatography-Mass Spectrometry (GC-MS). Food Chem..

[17] Grujić S.M., Stojanović G.S., Mitić V.D., Stankov-Jovanović V., Džamić A.M., Alimpić A.Z., Marin P.D. (2014). Evaluation of Antioxidant Activity of *Melittis melissophyllum* L. Extracts. Arch. Biol. Sci..

[18] Kaurinovic B., Popovic M., Vlaisavljevic S., Raseta M. (2011). Antioxidant Activities of *Melittis melissophyllum* L. (Lamiaceae). Molecules.

[19] Skrzypczak-Pietraszek E., Reiss K., Żmudzki P., Pietraszek J. (2018). Enhanced accumulation of harpagide and 8-*O*-acetyl-harpagide in *Melittis melissophyllum* L. agitated shoot cultures analyzed by UPLC-MS/MS. PLoS ONE.

[20] Górniak I., Bartoszewski R., Króliczewski J. (2018). Comprehensive review of antimicrobial activities of plant flavonoids. Phytochem. Rev..

[21] De Rossi L., Rocchetti G., Lucini L., Rebecchi A. (2025). Antimicrobial Potential of Polyphenols: Mechanisms of Action and Microbial Responses—A Narrative Review. Antioxidants.

[22] Periferakis A., Periferakis K., Badarau I.A., Petran E.M., Popa D.C., Caruntu A., Costache R.S., Scheau C., Caruntu C., Costache D.O. (2022). Kaempferol: Antimicrobial Properties, Sources, Clinical, and Traditional Applications. Int. J. Mol. Sci..

[23] Jan R., Khan M., Asaf S., Lubna, Asif S., Kim K.M. (2022). Bioactivity and Therapeutic Potential of Kaempferol and Quercetin: New Insights for Plant and Human Health. Plants.

[24] Harshad L., Jae-Seok K. (2023). Molecular Determinants of β -Lactam Resistance in Methicillin-Resistant *Staphylococcus aureus* (MRSA): An Updated Review. Antibiotics.

[25] Marciniak K., Tyczewska A., Grzywacz K. (2024). Genetics of Antibiotic Resistance in Methicillin-Resistant *Staphylococcus aureus* (MRSA). Biotechnologia.

[26] Nazzaro F., Fratianni F., De Martino L., Coppola R., De Feo V. (2013). Effect of Essential Oils on Pathogenic Bacteria. Pharmaceuticals.

[27] Salehi B., Venditti A., Sharifi-Rad M., Kręgiel D., Sharifi-Rad J., Durazzo A., Lucarini M., Santini A., Souto E.B., Novellino E. (2019). The Therapeutic Potential of Apigenin. Int. J. Mol. Sci..

[28] Xi M., Hou Y., Wang R., Ji M., Cai Y., Ao J., Shen H., Li M., Wang J., Luo A. (2022). Potential Application of Luteolin as an Active Antibacterial Composition in the Development of Hand Sanitizer Products. Molecules.

[29] Sezen Karaoğlan E., Hancı H., Koca M., Kazaz C. (2023). Some Bioactivities of Isolated Apigenin-7-*O*-Glucoside and Luteolin-7-*O*-Glucoside. Appl. Sci..

[30] Lou Z., Wang H., Rao S., Sun J., Ma C., Li J. (2012). P-Coumaric Acid Kills Bacteria through Dual Damage Mechanisms. Food Control.

[31] Kumar N., Goel N. (2019). Phenolic Acids: Natural Versatile Molecules with Promising Therapeutic Applications. Biotechnol. Rep..

[32] Bhattacharya M., Mandal S. (2025). Antibacterial Activity of Caffeic Acid from Plant Sources: A Review Based on in Silico, in Vitro and in Vivo Approaches. Microbe.

[33] Kernou O.-N., Azzouz Z., Madani K., Rijo P. (2023). Application of Rosmarinic Acid with Its Derivatives in the Treatment of Microbial Pathogens. Molecules.

[34] Saha P., Rahman F.I., Hussain F., Rahman S.M.A. (2022). Antimicrobial Diterpenes: Recent Development From Natural Sources. Front. Pharmacol..

[35] Saleh Mohammed A.A., Mickymaray S. (2020). Anti-Fungal Efficacy and Mechanisms of Flavonoids. Antibiotics.

[36] Dembinska K., Shinde A.H., Pejchalová M., Richert A., Brzezinska M.S. (2025). The Application of Natural Phenolic Substances as Antimicrobial Agents in Agriculture and Food Industry. Foods.

[37] Aćamović-Đoković G., Đukić D., Mandić L., Kalinić S., Bošković T. (2002). Antimicrobial Activity of the Petrol-Ether and Ethyl-Acetate Extracts of *Melilotus officinalis* (L.) Pall., Melilotus Albus Medic., and *Melittis melissophyllum* L. Lek. Sirovine.

[38] Abdel-Naime W.A., Fahim J.R., Fouad M.A., Kamel M.S. (2019). Antibacterial, Antifungal, and GC–MS Studies of Melissa Officinalis. S. Afr. J. Bot..

[39] Mabrouki H., Duarte C.M.M., Akretche D.E. (2018). Estimation of Total Phenolic Contents and In Vitro Antioxidant and Antimicrobial Activities of Various Solvent Extracts of *Melissa officinalis* L. Arab. J. Sci. Eng..

[40] Park Y.J., Baskar T.B., Yeo S.K., Arasu M.V., Al-Dhabi N.A., Lim S.S., Park S.U. (2016). Composition of Volatile Compounds and in Vitro Antimicrobial Activity of *Nine mentha* Spp. Springerplus.

[41] de Fátima Pires Carretto C., de Aguiar Almeida R.B., Furlan M.R., Jorge A.O.C., Junqueira J.C. (2010). Antimicrobial Activity of *Mentha piperita* L. against Candida Spp. Atividade Antimicrobiana de *Mentha piperita* L. *Sobre candida* Spp. Braz. Dent. Sci..

[42] Hudz N., Kobylinska L., Pokajewicz K., Horčinová Sedláčková V., Fedin R., Voloshyn M., Myskiv I., Brindza J., Wieczorek P.P., Lipok J. (2023). Mentha Piperita: Essential Oil and Extracts, Their Biological Activities, and Perspectives on the Development of New Medicinal and Cosmetic Products. Molecules.

[43] Sandasi M., Leonard C.M., Van Vuuren S.F., Viljoen A.M. (2011). Peppermint (*Mentha piperita*) Inhibits Microbial Biofilms in Vitro. S. Afr. J. Bot..

[44] Antolak H., Czyzowska A., Kregiel D. (2018). Activity of *Mentha Piperita* L. Ethanol Extract against Acetic Acid *Bacteria asaia* Spp. Foods.

[45] Budzyńska A., Wiȩckowska-Szakiel M., Sadowska B., Kalemba D., Rózalska B. (2011). Antibiofilm Activity of Selected Plant Essential Oils and Their Major Components. Pol. J. Microbiol..

[46] Yu H., Pei J., Qiu W., Mei J., Xie J. (2022). The Antimicrobial Effect of *Melissa officinalis* L. Essential Oil on Vibrio Parahaemolyticus: Insights Based on the Cell Membrane and External Structure. Front. Microbiol..

[47] Ranđelović M., Dimitrijević M., Otašević S., Stanojević L., Išljamović M., Ignjatović A., Arsić-Arsenijević V., Stojanović-Radić Z. (2023). Antifungal Activity and Type of Interaction of Melissa Officinalis Essential Oil with Antimycotics against Biofilms of Multidrug-Resistant Candida Isolates from Vulvovaginal Mucosa. J. Fungi.

[48] Yasir M., Dutta D., Willcox M.D.P. (2020). Activity of Antimicrobial Peptides and Ciprofloxacin against Pseudomonas Aeruginosa Biofilms. Molecules.

[49] Benedec D., Oniga I., Hanganu D., Vlase A.M., Ielciu I., Crisan G., Fit N., Niculae M., Bab T., Pall E. (2024). Revealing the Phenolic Composition and the Antioxidant, Antimicrobial and Antiproliferative Activities of Two Euphrasia Sp. Extracts. Plants.

[50] Ielciu I., Filip G.A., Sevastre-Berghian A.C., Bâldea I., Olah N.K., Burtescu R.F., Toma V.A., Moldovan R., Oniga I., Hanganu D. (2025). Effects of a *Rosmarinus officinalis* L. Extract and Rosmarinic Acid in Improving Streptozotocin-Induced Aortic Tissue Damages in Rats. Nutrients.

[51] Ielciu I., Filip G.A., Oniga I., Olah N.K., Bâldea I., Olteanu D., Burtescu R.F., Turcuș V., Sevastre-Berghian A.C., Benedec D. (2021). Oxidative Stress and DNA Lesion Reduction of a Polyphenolic Enriched Extract of Thymus Marschallianus Willd. In Endothelial Vascular Cells Exposed to Hyperglycemia. Plants.

[52] Simea S., Ielciu I., Hanganu D., Niculae M., Pall E., Burtescu R.F., Olah N., Cenariu M., Oniga I., Benedec D. (2023). Evaluation of the Cytotoxic, Antioxidative and Antimicrobial Effects of *Dracocephalum moldavica* L. Cultivars. Molecules.

[53] Buza V., Niculae M., Hanganu D., Pall E., Burtescu R.F., Olah N.K., Matei-Lațiu M.C., Vlasiuc I., Iozon I., Szakacs A.R. (2022). Biological Activities and Chemical Profile of Gentiana Asclepiadea and Inula Helenium Ethanolic Extracts. Molecules.

[54] Niculae M., Hanganu D., Oniga I., Burcă S.A., Tiperciuc B., Ielciu I., Pall E., Bab T., Burtescu R.F., Sava M.A. (2025). Agave Amica (Medik.) Thiede & Govaerts (Asparagaceae)—Insights into Its Valuable Phenolic Profile and In Vitro Antimicrobial, Antibiofilm, Antioxidative, and Antiproliferative Properties. Antibiotics.

[55] Olah N.-K., Osser G., Câmpean R.F., Furtuna F.R., Benedec D., Filip L., Raita O., Hanganu D. (2016). The Study of Polyphenolic Compounds Profile of Some *Rosmarinus officinalis* L. Extracts. Pak. J. Pharm. Sci..

[56] Matuschek E., Brown D.F.J., Kahlmeter G. (2014). Development of the EUCAST Disk Diffusion Antimicrobial Susceptibility Testing Method and Its Implementation in Routine Microbiology Laboratories. Clin. Microbiol. Infect..

[57] CSLI—Clinical and Laboratory Standards Institute (2012). Methods for Dilution Antimicrobial Susceptibility Tests for Bacteria That Grow Aerobically.

[58] Wang X., Liu M., Yu C., Li J., Zhou X. (2023). Biofilm Formation: Mechanistic Insights and Therapeutic Targets. Mol. Biomed..

[59] Schulze A., Mitterer F., Pombo J.P., Schild S. (2021). Biofilms by Bacterial Human Pathogens: Clinical Relevance—Development, Composition and Regulation—Therapeutical Strategies. Microb. Cell.

[60] Malinovská Z., Čonková E., Váczi P. (2023). Biofilm Formation in Medically Important Candida Species. J. Fungi.

