# Supplementary material for: Integrated Chemical Profiling and Biological Assessment of Melittis melissophyllum L. (Lamiaceae): Antioxidant, Antimicrobial and Cytotoxic Activities
Source: Antibiotics (Basel). 2026 Jul 31;15(8):742. doi: 10.3390/antibiotics15080742 (PMC13509644; doi:10.3390/antibiotics15080742)
Supplement: Supplementary file 1 [file antibiotics-15-00742-s001.zip › antibiotics-4437529-supplementary.pdf]

antibiotics

MDPI

Supplementary materials

Integrated Chemical Profiling and Biological Assessment of *Melittis melissophyllum* L. (Lamiaceae): Antioxidant, Antimicrobial and Cytotoxic Activities

Daniela Benedec [1], Irina Ielciu [2,*], Emoke Pall [3], Adriana Dalila Criste [4], Adriana Cristina Urcan [4], Ilioara Oniga [1], Timea Henrietta Dezső (Bab) [1], Ramona Flavia Burtescu [5] and Daniela Hanganu [1]

1 Department of Pharmacognosy, Faculty of Pharmacy, "Iuliu Hat,ieganu" University of Medicine and Pharmacy, 400010 Cluj-Napoca, Romania; dbenedec@umfcluj.ro (D.B.); ioniga@umfcluj.ro (I.O.); bab.timea.henrietta@elearn.umfcluj.ro (T.H.D.); dhanganu@umfcluj.ro (D.H.)
2 Department of Pharmaceutical Botany, Faculty of Pharmacy, "Iuliu Hat,ieganu" University of Medicine and Pharmacy, 400337 Cluj-Napoca, Romania
3 Department of Clinical Sciences, University of Agricultural Sciences and Veterinary Medicine Cluj-Napoca, 400001 Cluj-Napoca, Romania; emoke.pall@usamvcluj.ro
4 Department of Microbiology and Immunology, Faculty of Animal Science and Biotechnologies, University of Agricultural Sciences and Veterinary Medicine Cluj-Napoca, 400001 Cluj-Napoca, Romania; adriana.criste@usamvcluj.ro (A.D.C.); adriana.urcan@usamvcluj.ro (A.C.U.)
5 PlantExtrakt Ltd., 407059 Cluj-Napoca, Romania; ramona.burtescu@plantextrakt.ro
* Correspondence: irina.ielciu@umfcluj.ro

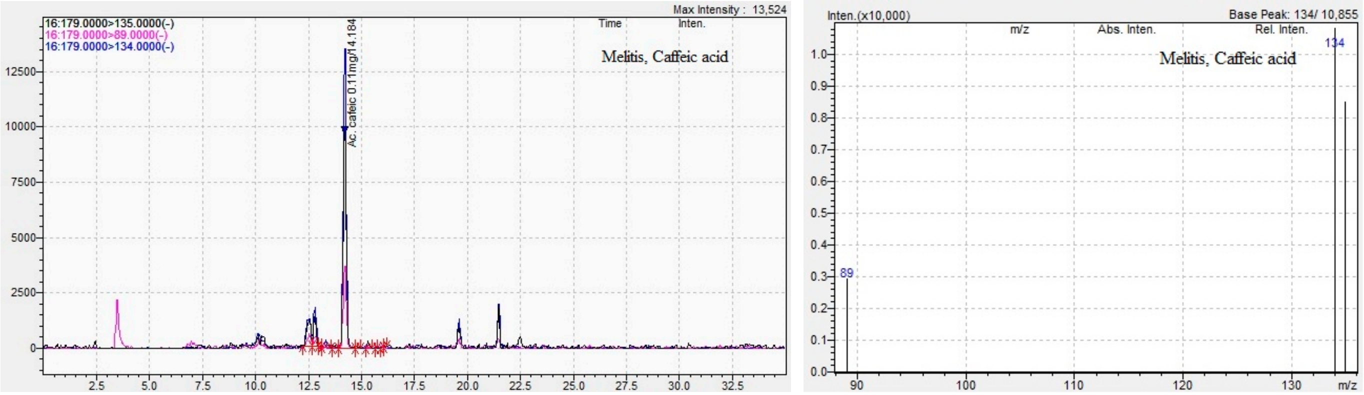

Figure S1. LC-MS chromatogram and mass fragmentation pattern obtained for the identification of caffeic acid in the *M. melissophyllum* extract

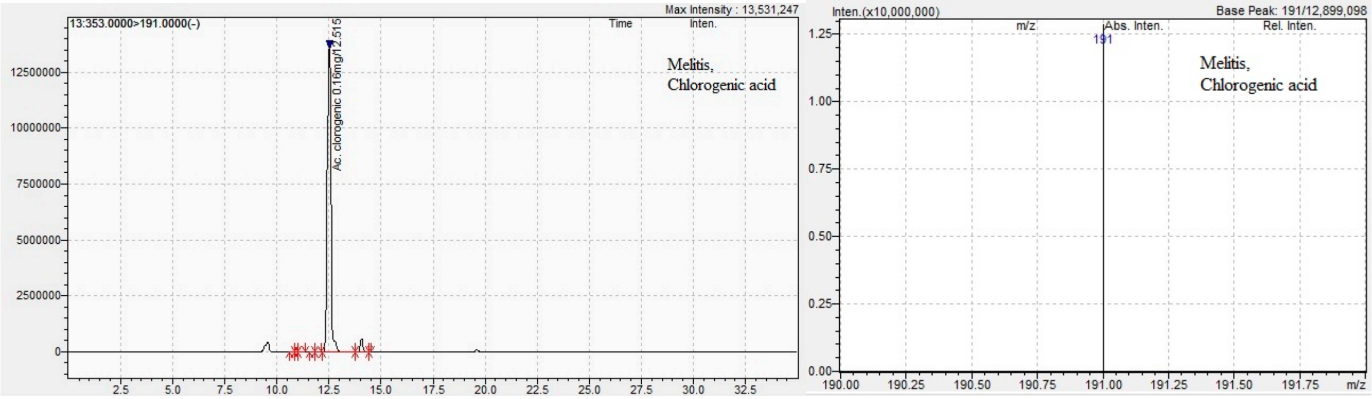

Figure S2. LC-MS chromatogram and mass fragmentation pattern obtained for the identification of chlorogenic acid in the *M. melissophyllum* extract

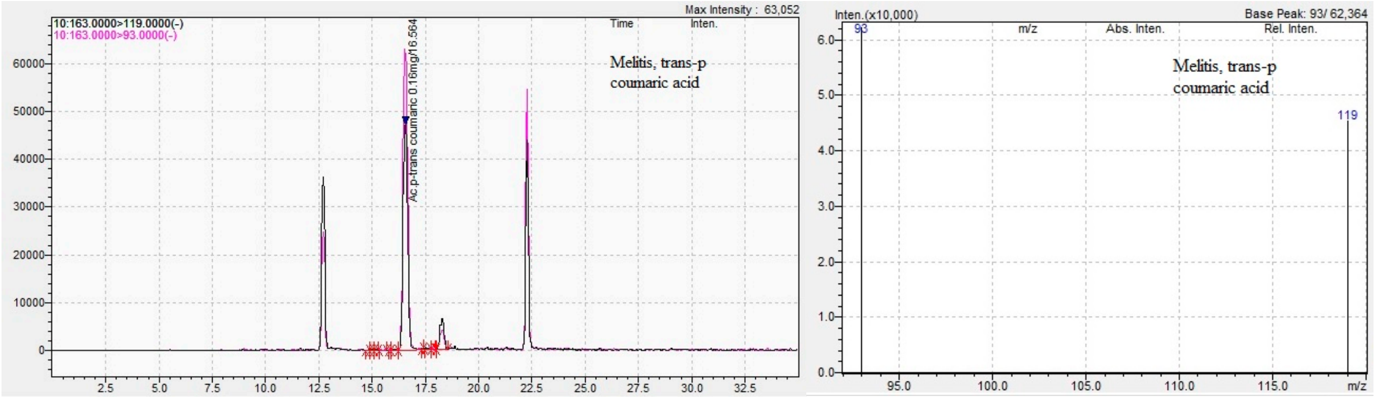

Figure S3. LC-MS chromatogram and mass fragmentation pattern obtained for the identification of trans-*p*-coumaric acid in the *M. melissophyllum* extract

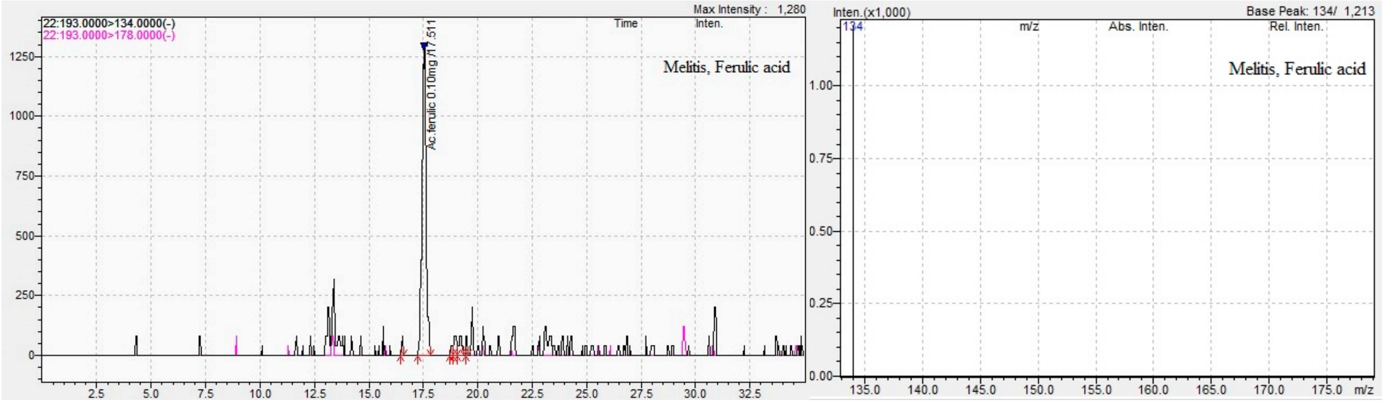

Figure S4. LC-MS chromatogram and mass fragmentation pattern obtained for the identification of ferulic acid in the *M. melissophyllum* extract

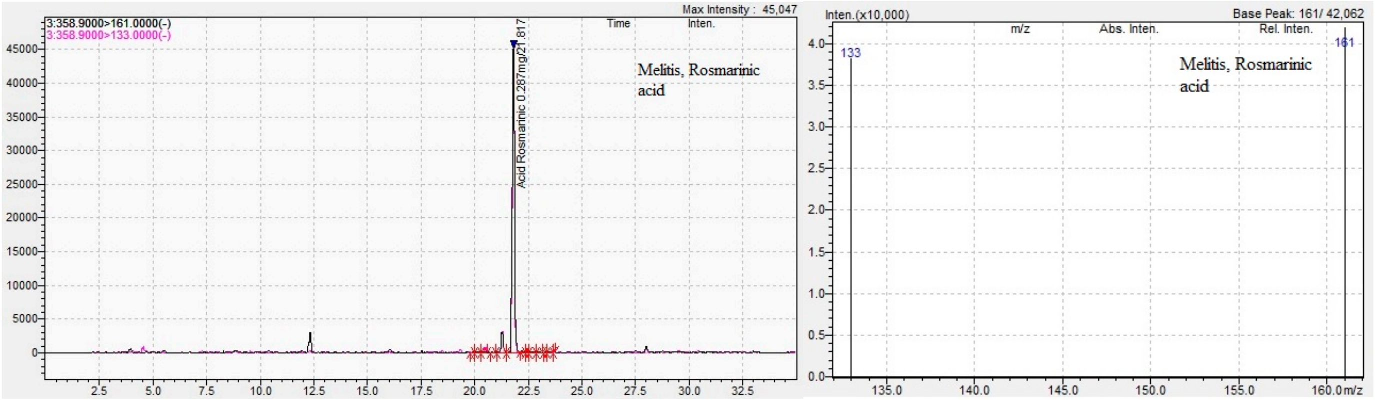

Figure S5. LC-MS chromatogram and mass fragmentation pattern obtained for the identification of rosmarinic acid in the *M. melissophyllum* extract

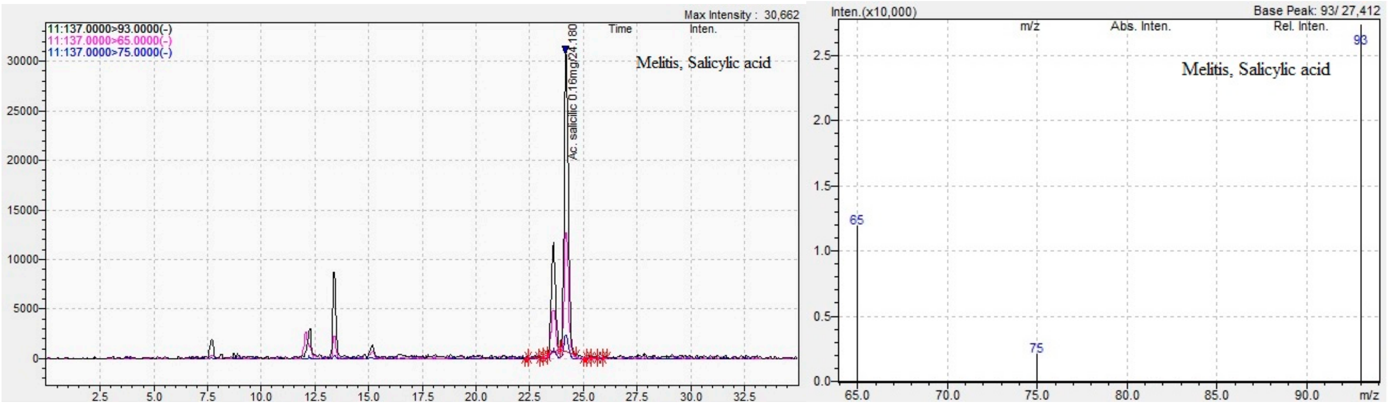

Figure S6. LC-MS chromatogram and mass fragmentation pattern obtained for the identification of salicylic acid in the *M. melissophyllum* extract

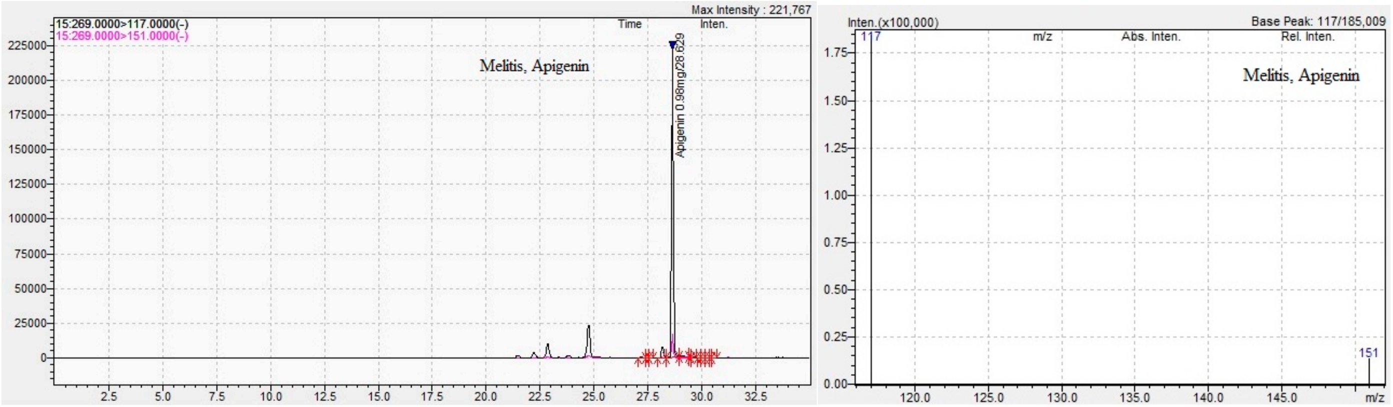

Figure S7. LC-MS chromatogram and mass fragmentation pattern obtained for the identification of apigenin in the *M. melissophyllum* extract

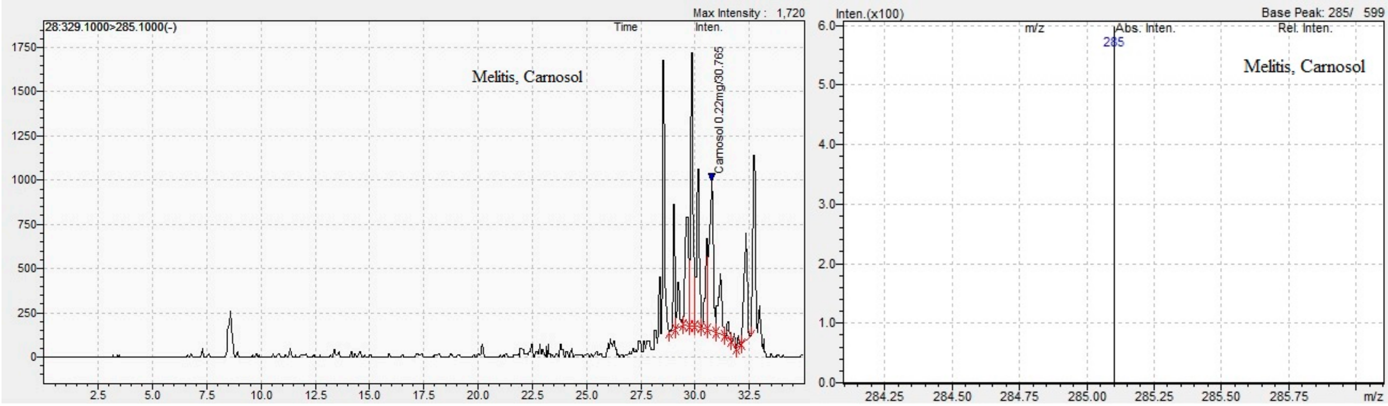

Figure S8. LC-MS chromatogram and mass fragmentation pattern obtained for the identification of carnosol in the *M. melissophyllum* extract

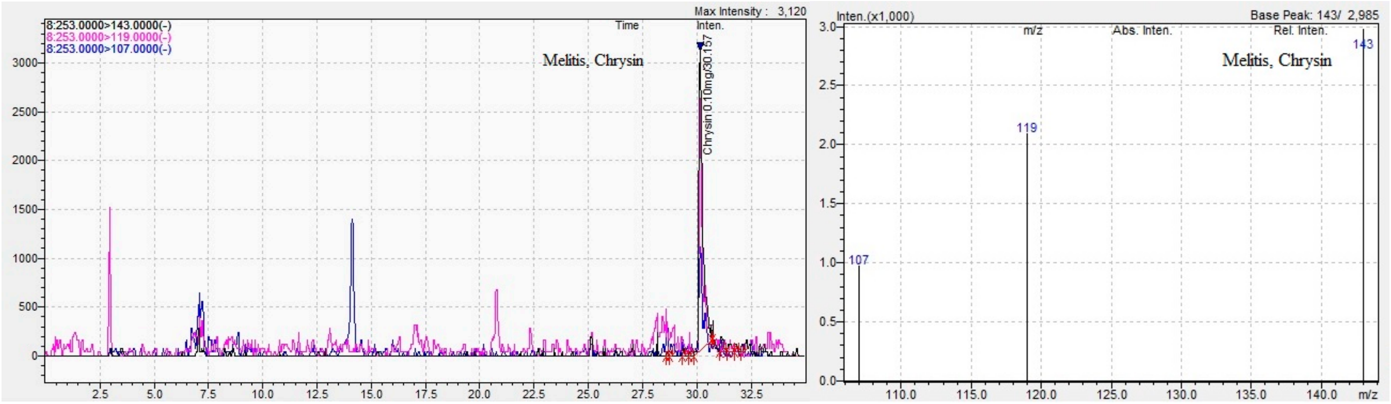

Figure S9. LC-MS chromatogram and mass fragmentation pattern obtained for the identification of chrysin in the *M. melissophyllum* extract

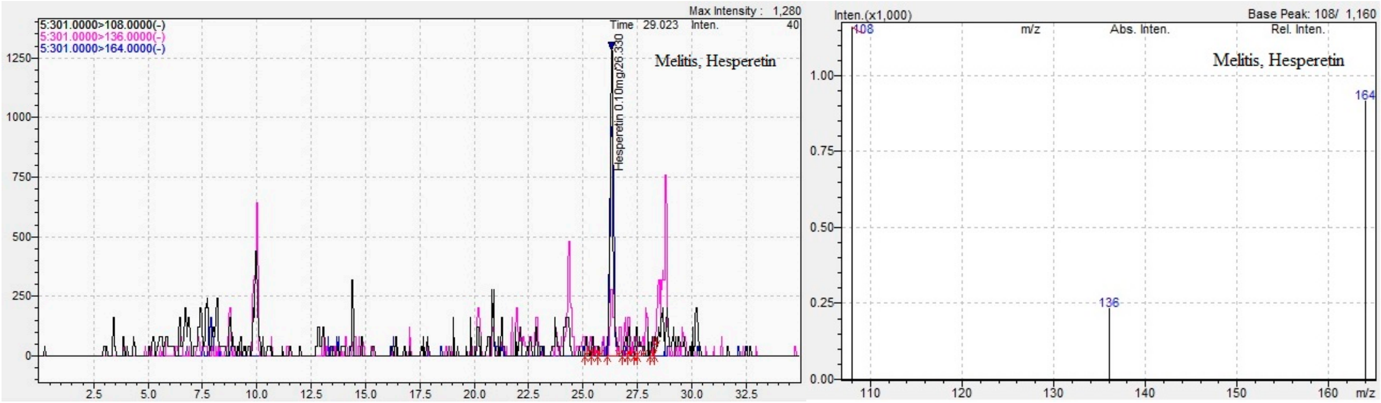

Figure S10. LC-MS chromatogram and mass fragmentation pattern obtained for the identification of hesperetin in the *M. melisso-phyllum* extract

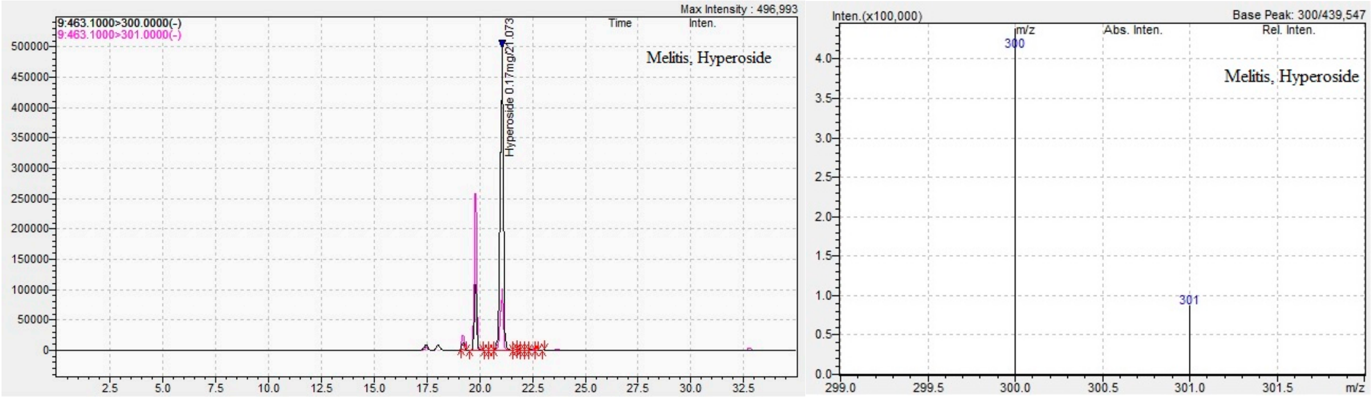

Figure S11. LC-MS chromatogram and mass fragmentation pattern obtained for the identification of hyperoside in the *M. melisso-phyllum* extract

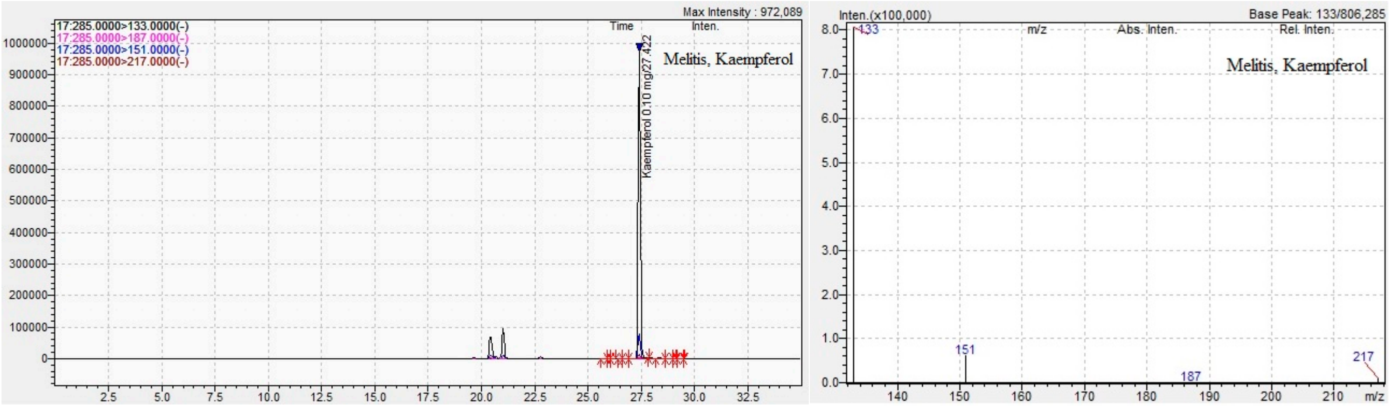

Figure S12. LC-MS chromatogram and mass fragmentation pattern obtained for the identification of kaempferol in the *M. melissophyllum* extract

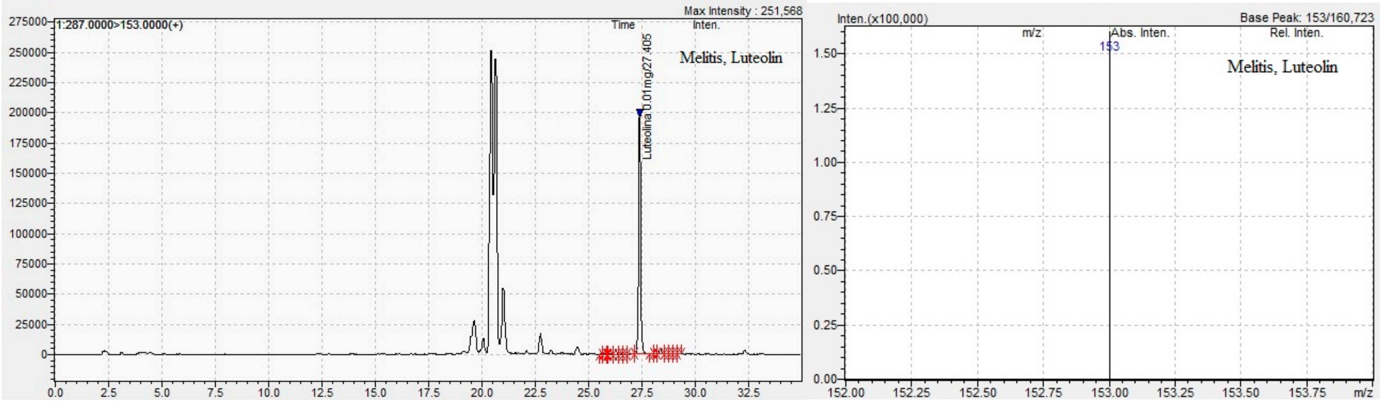

Figure S13. LC-MS chromatogram and mass fragmentation pattern obtained for the identification of luteolin in the *M. melissophyllum* extract

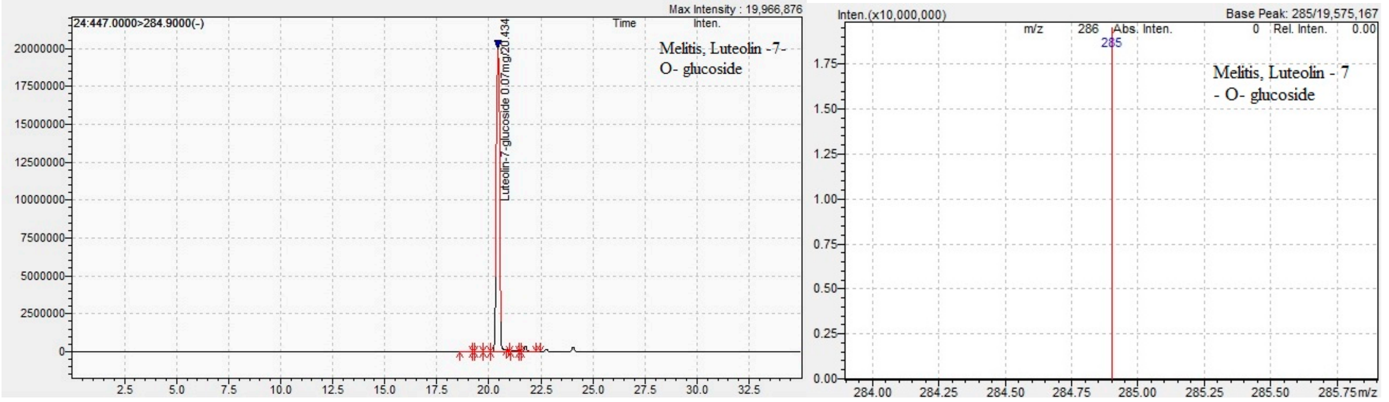

Figure S14. LC-MS chromatogram and mass fragmentation pattern obtained for the identification of luteolin-7-*O*-glucoside in the *M. melissophyllum* extract

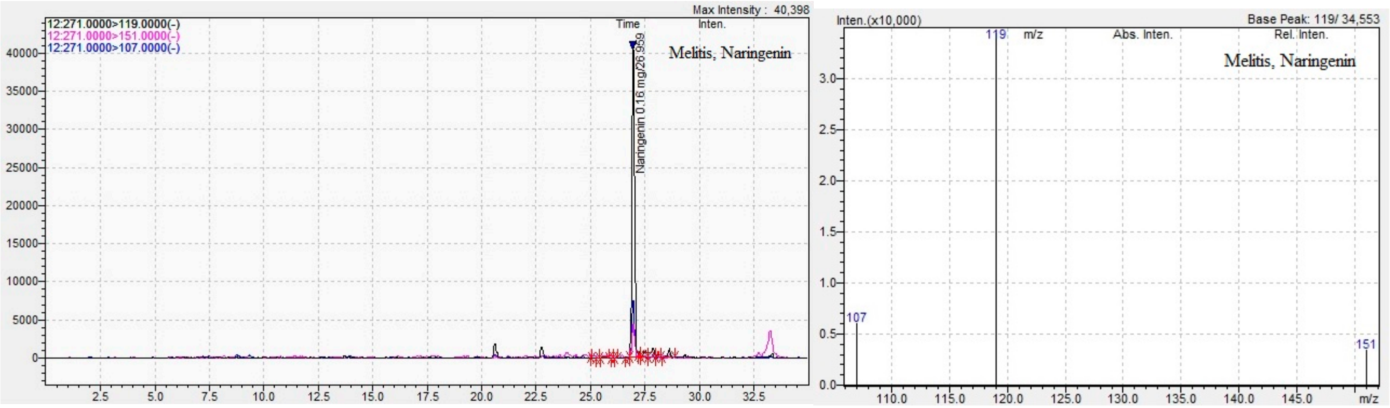

Figure S15. LC-MS chromatogram and mass fragmentation pattern obtained for the identification of naringenin in the *M. melissophyllum* extract

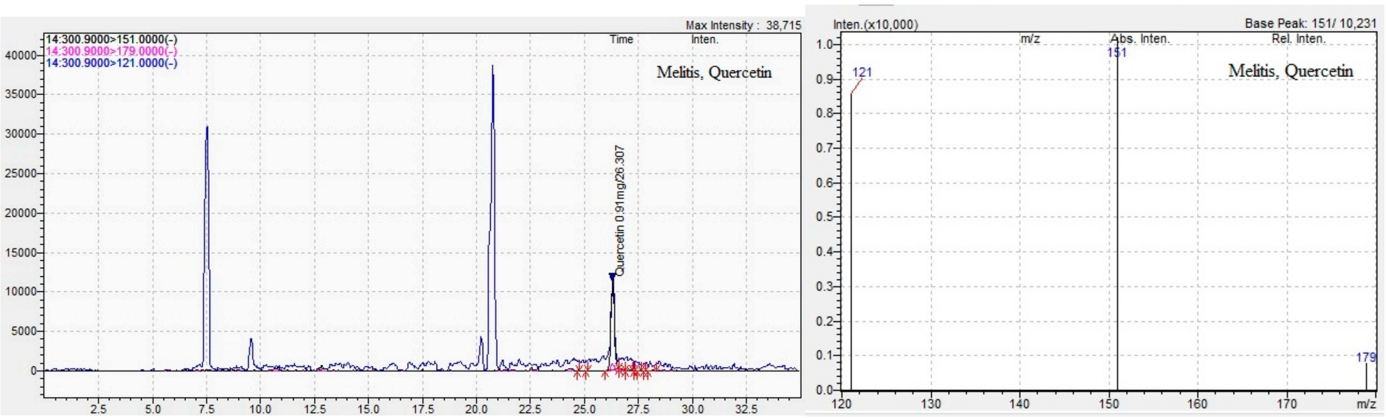

Figure S16. LC-MS chromatogram and mass fragmentation pattern obtained for the identification of quercetin in the *M. melissophyllum* extract

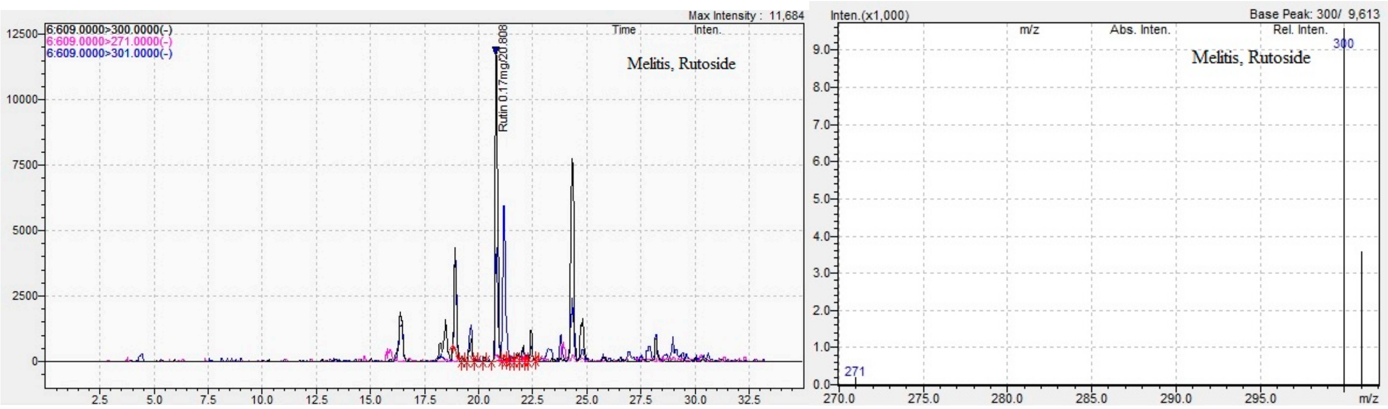

Figure S17. LC-MS chromatogram and mass fragmentation pattern obtained for the identification of rutoside in the *M. melissophyllum* extract
